# A simple and efficient method for the preparation of 5-hydroxy-3-acyltetramic acids

**DOI:** 10.3762/bjoc.11.37

**Published:** 2015-03-06

**Authors:** Johanna Trenner, Evgeny V Prusov

**Affiliations:** 1 Department of Medicinal Chemistry, Helmholtz Centre for Infection Research, Inhoffenstr. 7, 38124 Braunschweig, Germany

**Keywords:** heterocyclic chemistry, hydroxylation, natural products, tetramic acids

## Abstract

Oxidation of the bisenolates of 3-acyltetramic acid to the corresponding 5-hydroxylated compounds using molecular oxygen is reported. The deprotection of the resulting compounds was also achieved.

## Findings

**Figure 1:**
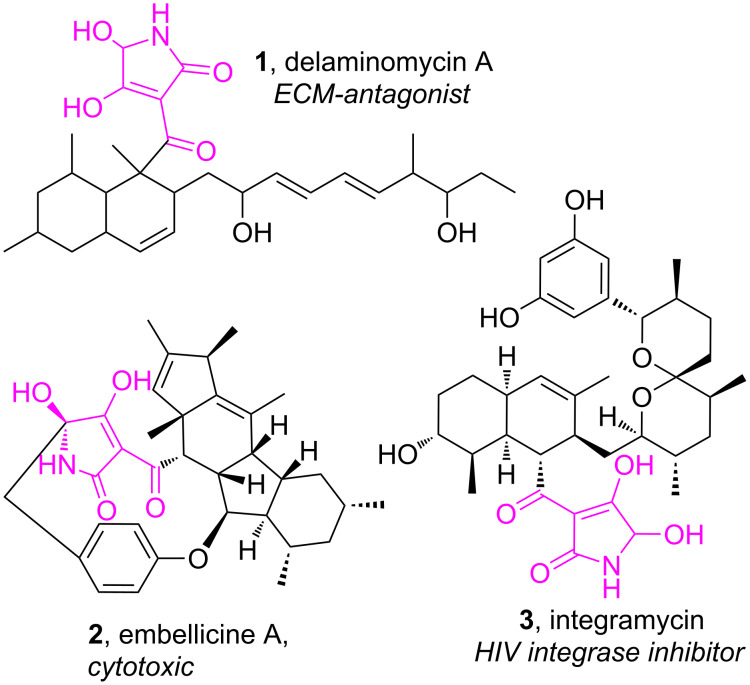
Naturally occurring 5-hydroxylated 3-acyltetramic acids.

5-Hydroxy-3-acyltetramic acid is an unusual structural element which is found in the molecules of such biologically active natural products as delaminomycin A [1], embellicin A [2] and integramycin [3] (Figure 1).

Additionally, 5-heterosubstituted-3-acyltetramic acids were recently identified by Moloney as perspective lead structures for the development of novel antibacterial compounds [4]. Intrigued by their potential antibiotic properties, we were interested to investigate their activities in various assays but the literature survey showed that synthetic approaches to 5-hydroxylated 3-acyltetramic acids are essentially non-existent, although M. Coster reported the synthesis of 5-hydroxytetramic acid ethers by chemoselective reduction of the corresponding maleimides [5] and oxidation of pyrrolinones was reported by Clayden [6]. Therefore, we decided to develop a simple method to produce these interesting compounds.

**Scheme 1:**
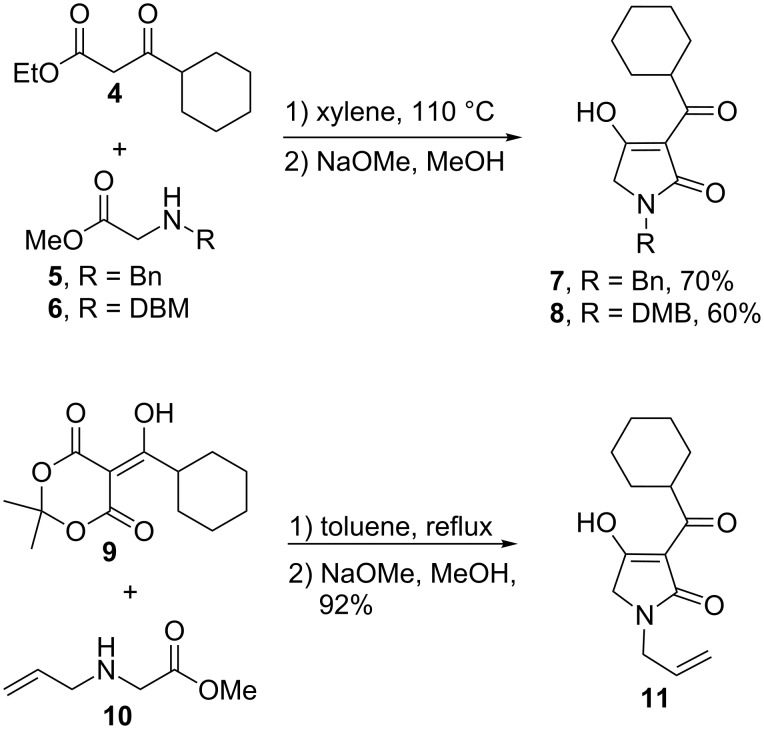
Synthesis of model tetramic acids.

At first we prepared three nitrogen-protected 3-acyltetramic acids (**7**–**9**) according to the Matsuo [7] variant of Lacey–Dieckmann [8] condensation (Scheme 1) [9–10]. Reaction of either β-ketoester **4** or Meldrum’s acid derivative **9** with suitably protected glycine esters (**5**,**6**,**10**), followed by base-induced condensation furnished the desired tetramic acid model compounds as crystalline solids after treatment with a small quantity of methanol. Alternatively, purification of these compounds can be readily achieved by preparative HPLC, whereas all attempts to perform the standard flash column chromatography on silica gel or aluminum oxide resulted in complete decomposition of the material.

With the substrates in hands, we initially investigated the use of Davis oxaziridine chemistry (Table 1, entries 1 and 2) [11]. Deprotonation of **7** with an excess of LDA followed by treatment of the resulting bisenolate with camphoryl-based oxaziridine reagent **13** provided the desired product in 27% yield. Similar results were obtained when 3-phenyl-2-(phenylsulfonyl)oxaziridine (**14**) was employed, but product **15** from the concomitant reaction of bisenolate addition to *N*-sulfonimine byproduct was also isolated in 15% yield from this reaction. Some peroxide-based electrophilic oxidants (Table 1, entries 3–5) were also briefly tested, but were found to produce rather mediocre yields of hydroxylated compounds. Attempted oxidations with Oxone or air gave no product at all.

**Table 1:** Initial study of oxidation of tetramic acid **7**.

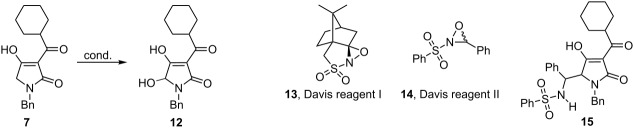				

entry	base	solvent	reagent, *T*, *t*	yield

1	LDA (2.5 equiv)	THF	Davis reagent I, **13** (1.5 equiv), 2 h, −78 °C	27%
2	LDA (2.5 equiv)	THF	Davis reagent II, **14** (1.5 equiv), 30 min, −78 °C	28%^a^
3	LDA (2.5 equiv)	THF	(BzO)_2_ (1.5 equiv), 1.5 h, −78 °C	1%
4	LDA (2.5 equiv)	THF	*t*-BuOOBz (1.5 equiv), 1.5 h, −78 °C	8%^b^
5	LDA (2.5 equiv)	THF	(*t*-BuO)_2_ (1.5 equiv), 1.5 h, −78 °C	0%
6	–	H_2_O	O_2_, rt, 14 d	0%
7	–	MeOH/H_2_O	Oxone^®^, rt, 1 d	0%

^a^15% of **15** were also isolated. ^b^Yield of benzoate derivative.

Our efforts to improve the yield of the desired product by variation of reaction conditions were rather fruitless, and therefore, our attention was turned to the alternative oxidation of enolates with molecular oxygen in the presence of triethyl phosphite as originally described by Hartwig [12–15]. Application of these conditions resulted in a clean conversion to the 5-hydroxy-3-acyltetramic acid but again, the isolated yield of the product was rather moderate. Switching to KHMDS as a base dramatically increased the yield and with further optimization of the reaction time and dilution a 78% yield (brsm) of the hydroxylated compound was achieved (Table 2). Discoloration of the bisenolate solution was usually observed within 5 minutes and the reaction was essentially complete in 20 minutes according to TLC analysis. Oxidation reactions performed in polar aprotic solvents, such as DMPU and DMF, gave significantly lower yields compared to THF. As with the parent tetramic acids, isolation and purification of the hydroxylated derivatives was only possible by means of preparative HPLC.

**Table 2:** Condition optimization for hydroxylation of tetramic acid **7**.

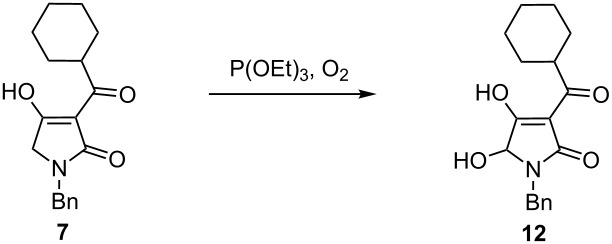				

entry	base	solvent	*t*, *T*	yield^a^

1	LDA	THF	30 min, −78 °C	27%
2	KO*t*-Bu	THF	3 h, −78 °C; 14 h, rt	10% (14%)
3	LiHMDS	THF	2 h, −78 °C	51% (59%)
4	NaHMDS	THF	2 h, −78 °C	44% (51%)
5	KHMDS	THF	35 min, −78 °C	74% (77%)
6	KHMDS	THF	10 min, −78 °C	72% (78%)
7	KHMDS	DME	2 h 15 min, −78 °C	11% (18%)
8	KHMDS	DMPU	1 h 15 min, −78 °C	20% (36%)
9	KHMDS	DMF	2 h, −78 °C	32% (48%)

^a^Yields in parentheses are based upon recovered starting material.

Similar results were obtained for the *N*-DMB and *N*-allyl protected tetramic acids, though the best yield of compound **16** was obtained when LDA was employed as a base (Table 3).

**Table 3:** Oxidation of tetramic acids **8** and **11**.

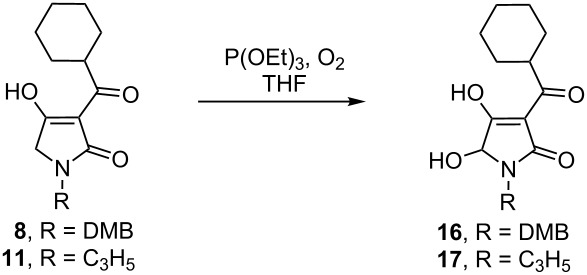				

entry	comp.	base	*t*, *T*	yield^a^

1	**8**	KHMDS	10 min, −78 °C	49% (61%)
2	**8**	KHMDS	25 min, −78 °C	55% (69%)
3	**8**	KHMDS	40 min, −78 °C	62% (69%)
4	**8**	LDA	5 min, −78 °C	47% (55%)
5	**8**	LDA	5 min, −78 °C	56% (62%)
6	**11**	KHMDS	4h, −78 °C to rt	42% (44%)

^a^Yields in parentheses are based upon recovered starting material.

**Scheme 2:**
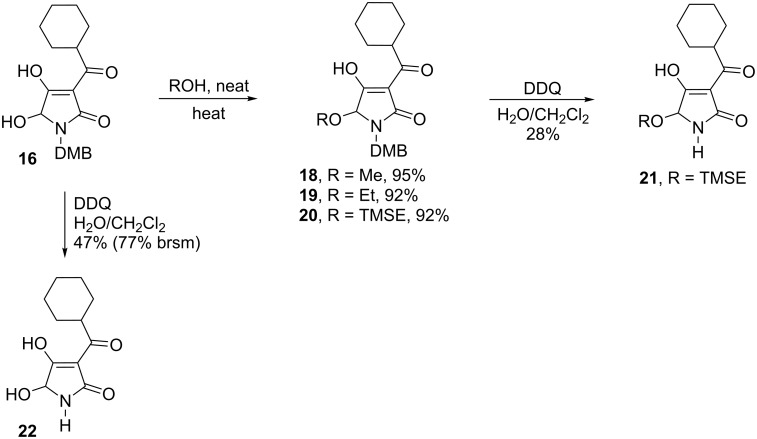
Synthesis of hemiaminal ethers and deprotection of the tetramic acids.

The obtained hydroxylated tetramic acids can readily be transformed to hemiaminal ethers by simple heating them with the corresponding alcohol (Scheme 2). Etherification with methanol, ethanol and 2-trimethylsilylethanol produced the hemiaminal ethers in almost quantitative yields. We next investigated the deprotection of the so obtained hydroxylated derivatives. Several attempts to remove the benzyl group under various catalytic hydrogenation conditions failed entirely. Cleavage of the DMB group with TFA [16] was also tried, but brought no success and extensive formation of polymerization products of the corresponding iminium ion was observed. Oxidative cleavage of the DMB group with CAN [17] produced no product either. Successful removal of the DMB-protecting group from hydroxylated tetramic acid **16** and (trimethylsilyl)ethyl hemiaminal **19** was achieved upon treatment with an excess of DDQ in wet DCM. Treatment of the *N*-allyl-protected compound using catalytic amounts of Pd trifluoroacetate/dppp [18] gave no conversion.

In summary, we have developed a simple and efficient method for the synthesis of 5-hydroxy-3-acyltetramic acids by oxidation of the corresponding bisenolates with molecular oxygen. We have also investigated the cleavage of various protecting groups from the nitrogen of tetramic acids. Application of this methodology to a broader scope of substrates as well as to the total synthesis of natural products is currently underway in our laboratory and will be reported in due course.

## Experimental

**General procedure for the oxidation of tetramic acids:** Under an argon atmosphere, KHMDS (1 M in THF, 2.5 equiv) was added at −78 °C to a solution of 3-cyclohexancarbonyltetramic acid (1.0 equiv) in dry THF (0.2 M) and stirred for 25 min. After the addition of P(OEt)_3_ (2.0 equiv), oxygen from rubber balloon (predried by passing through a tube filled with P_4_O_10_) was passed through the bright yellow reaction mixture until full conversion (decolorizing and TLC control). Saturated NH_4_Cl solution (3 mL) was added and the reaction mixture was extracted with EtOAc. After evaporation of the solvent the residue was extracted with SPE (H_2_O, then MeCN) and the organic phase was purified with preparative HPLC (Machery Nagel, Nucleodur VP250/21 C18 Gravity, 5 µm; 280 nm; 90:10 MeCN/(H_2_O + 1% formic acid); 10 mL/min) to give the corresponding 5-hydroxy-3-cyclohexanecarbonyl tetramic acid as a white solid.

## Supporting Information

File 1 Experimental procedures.

File 2 Copies of NMR spectra.
