# Supplementary material for: A simple and efficient method for the preparation of 5-hydroxy-3-acyltetramic acids
Source: Beilstein J Org Chem. 2015 Mar 6;11:323–7. doi: 10.3762/bjoc.11.37 (PMC4362042; doi:10.3762/bjoc.11.37)
Supplement: File 1 — Experimental procedures. [file Beilstein_J_Org_Chem-11-323-s001.pdf]

Supporting Information

for

A simple and efficient method for the preparation of 5-hydroxy-3-acyltetramic acids

Johanna Trenner[1], Evgeny V. Prusov*[1]

Address: [1]Department of Medicinal Chemistry, Helmholtz Centre for Infection Research, Inhoffenstr. 7, 38124 Braunschweig, Germany

Email: Evgeny V. Prusov - evgeny.prusov@helmholtz-hzi.de

* Corresponding author

Experimental procedures

General remarks

Air-sensitive reactions were carried out using standard Schlenk technique with argon as inert gas and commercially available absolute solvents. Yields that are based on recovered starting material are indicated with (brsm). Aluminum sheets covered with silica gel (Si 60 F_{254}, Merck) were used for analytical TLC. Spots were detected using UV absorption at 254 nm and staining with cerium(IV) sulfate/phosphomolybdic acid in sulfuric acid or with vanillin/sulfuric acid/acetic acid in methanol followed by charring. Preparative HPLC was performed on a VarioPrep Nucleodur C18 Gravity column (250 x 21 mm, 5 µm, Machery Nagel). HPLC solvent A refers to a mixture of 95:5 (v/v) H_2O/MeCN + 1% formic acid which were filtrated over a polyamide membrane filter (0.45 µm, Whatman). HPLC solvent B refers to MeCN (HPLC grade). NMR spectra were recorded on a Bruker AMX 500 or AMX 700 spectrometer. Chemical shifts are given in [ppm] and are relative to residual $CDCl_3$ (δ_H = 7.25, δ_C = 77.0) or CD_3CN (δ_H = 1.94, δ_C = 1.3, 118.3). Coupling constants *J* are given in [Hz]. HRMS–ESI spectra were measured on a Bruker Maxis (ESI-TOF-MS).

Condensation of 3-acyltetramic acids

N-Benzyl-3-(cyclohexanecarbonyl)tetramic acid (7)

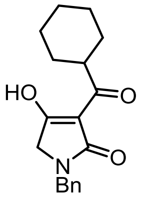

Under an argon atmosphere, a solution of methyl *N*-benzyl glycinate (**5**) (1.02 equiv, 0.54 mmol, 96 mg) and ethyl 3-cyclohexyl-3-oxopropanoate (**4**) (1.0 equiv, 0.53 mmol, 104 mg) in dry xylene (0.80 M, 0.66 mL) was heated to 130 °C (bath temperature) for 40 min. After cooling to room temperature the reaction mixture was transferred via syringe to freshly prepared NaOMe (from NaH (1.13 equiv, 0.60 mmol, 24 mg) and dry MeOH (0.50 mL)) and stirred at room temperature for 15 min. The reaction mixture was then added to a vigorously stirred mixture of 1 M HCl (5 mL) and EtOAC (6 mL). The organic phase was washed with brine (10 mL) and concentrated under reduced pressure to give the title compound as a white solid (109.3 mg, 0.37 mmol, 70% yield).

R_f = 0.08 (PE/EtOAc 2:1).

t_R = 11.6 min (280 nm, 10:90 A:B, 10 mL/min).

^{1}H NMR (500 MHz, $CDCl_3$): δ [ppm] = 7.37-7.27 (m, 5H), 7.25 (s, 0.4H, minor tautomer), 7.24 (s, 1H), 4.60 (s, 0.4H, minor tautomer), 4.59 (s, 2H), 3.71 (s, 0.4H, minor tautomer), 3.58 (s, 2H), 3.38 (tt, *J* = 3.38, 11.68 Hz, 1H), 1.90-1.67 (m, 7H), 1.51 (dq, *J* = 3.41, 17.19 Hz, 3H), 1.37 (tq, *J* = 3.45, 12.95 Hz, 3H), 1.31-1.17 (m, 3H).

^{13}C NMR (125 MHz, $CDCl_3$): δ [ppm] = 191.7, 191.1, 173.9, 135.4, 129.0, 128.3, 100.3, 55.0, 45.5, 40.9, 28.7, 25.5.

HR-ESI-MS for $[M+H]^+$ $C_{18}H_{22}NO_3$: calc. 300.1594, found 300.1593. HR-ESI-MS for $[M+Na]^+$ $C_{18}H_{21}NNaO_3$: calc. 322.1414, found 322.1410.

N-(2,4-Dimethoxybenzyl)-3-(cyclohexanecarbonyl)tetramic acid (8)

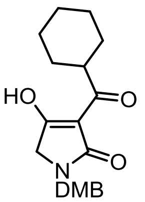

Under an argon atmosphere, a solution of methyl *N*-dimethoxybenzyl glycinate (**6**) (1.04 equiv, 1.05 mmol, 250 mg) and ethyl 3-cyclohexyl-3-oxopropanoate (**4**) (1.0 equiv, 1.01 mmol, 200 mg) in dry xylene (0.76 M, 1.32 mL) was heated to 130 °C (bath temperature) for 1 h. After cooling to room temperature the reaction mixture was transferred via syringe to freshly prepared NaOMe (from NaH (1.06 equiv, 1.07 mmol, 43 mg) and dry MeOH (1 mL)) and stirred at room temperature for 20 min. The reaction mixture was then added to a vigorously stirred mixture of 1 M HCl (10 mL) and EtOAC (12 mL). The organic phase was washed with H_2O (20 mL) and concentrated under reduced pressure. Recrystallization from MeOH delivered the title compound as a bright yellow solid (225 mg, 0.63 mmol, 60% yield).

R_f = 0.54 (EtOAc).

t_R = 11.2 min (280 nm, 10:90 A:B, 10 mL/min).

Mixture of tautomers 1:0.15.

^{1}H NMR (500 MHz, $CDCl_3$): δ [ppm] = 7.18 (d, *J* = 8.90 Hz, 0.15H, minor tautomer), 7.16 (d, *J* = 8.80 Hz, 1H), 6.46-6.43 (m, 2.3H, with minor tautomer), 4.54 (s, 0.3H, minor tautomer), 4.53 (s, 2H), 3.80 (s, 3.4H, with minor tautomer), 3.79 (s, 3.5H, with minor tautomer), 3.62 (s, 2H), 3.36 (tt, *J* = 3.39, 11.86 Hz, 1H), 1.82-1.66 (m, 6H), 1.55-1.45 (m, 2H), 1.42-1.30 (m, 2H), 1.29-1.16 (m, 2H).

^{13}C NMR (125 MHz, $CDCl_3$): δ [ppm] = 191.8, 191.3, 173.7, 161.0, 158.7, 131.4, 116.1, 104.4, 100.5, 98.6, 55.8, 55.4, 55.4, 40.8, 40.0, 28.7, 25.7, 25.5.

HR-ESI-MS for $[M+H]^+$ $C_{20}H_{26}NO_5$: calc. 360.1806, found 360.1803. HR-ESI-MS for $[M+Na]^+$ $C_{20}H_{25}NNaO_5$: calc. 382.1625, found 382.1621.

N-Allyl-3-(cyclohexanecarbonyl)tetramic acid (11)

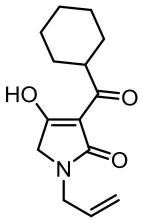

Under an argon atmosphere, a solution of methyl *N*-allyl glycinate (**10**) (0.26 mL, 254 mg, 1.97 mmol, 1.0 equiv) and 5-(cyclohexyl(hydroxy)methylene)-2,2-dimethyl-1,3-dioxane-4,6-dione (**9**, 500 mg, 1.97 mmol, 1.0 equiv) in dry toluene (5 mL, 0.4 M) was stirred at 105 °C (bath temperature) for 1 h. After cooling to room temperature the reaction mixture was added to freshly prepared NaOMe (from NaH (60% in mineral oil, 83 mg, 2.08 mmol, 1.06 equiv) and dry MeOH (2 mL)) and stirred for 40 min. The reaction mixture was poured into a vigorously stirred mixture of 1 M HCl (20 mL) and EtOAc (24 mL) and the organic phase was washed with water (20 mL) and concentrated under reduced pressure to give the title compound as yellow resin (452 mg, 1.81 mmol, 92% yield). Further purification can be performed with preparative HPLC (210 nm, 10:90 A:B, 9 mL/min, t_R = 9.6 min).

Mixture of tautomers 1:0.3.

^{1}H NMR (500 MHz, $CDCl_3$): δ [ppm] = 5.76 (tdd, *J* = 6.16, 10.19, 17.12 Hz, 1.3H), 5.28-5.18 (m, 3H), 4.04 (td, *J* = 1.38, 5.95 Hz, 2.4H), 3.81 (s, 0.4H, minor tautomer), 3.68 (s, 2H), 3.55 (tt, *J* = 3.24, 11.72 Hz, 0.3H, minor tautomer), 3.39 (tt, *J* = 3.34, 11.69 Hz, 1H), 1.87-1.68 (m, 7H), 1.57-1.46 (m, 2.5H), 1.38 (tq, *J* = 3.34, 12.92 Hz, 3H), 1.23 (tq, *J* = 3.09, 12.66 Hz, 3H).

^{13}C NMR (125 MHz, $CDCl_3$): δ [ppm] = 191.7, 191.2, 173.8, 132.4 (minor tautomer), 131.5, 119.0, 118.4 (minor tautomer), 100.3, 55.1, 52.0 (minor tautomer), 44.0, 40.8, 28.6, 25.6, 25.5.

HR-ESI-MS for $[M+H]^+$ $C_{14}H_{20}NO_3$: calc. 250.1438, found 250.1435. HR-ESI-MS for $[M+Na]^+$ $C_{14}H_{19}NNaO_3$: calc. 272.1257, found 272.1253.

Oxidation with Davis oxaziridines

Under an argon atmosphere, methyl lithium (1.19 M in Et_2O, 0.35 mL, 0.42 mmol, 2.5 equiv) was added dropwise at −78 °C to a solution of diisopropyl amine (59 µL, 42.3 mg, 0.42 mmol, 2.5 equiv) in dry THF (0.67 mL). After 1 h, a solution of *N*-benzyl-3-(cyclohexanecarbonyl)tetramic acid (**7**) (50.0 mg, 0.17 mmol, 1.0 equiv) in dry THF (0.33 mL) was added at −78 °C to give a cloudy orange solution which was stirred for 20 min. Then a solution of Davis reagent I (**13**, 57.4 mg, 0.25 mmol, 1.5 equiv) in dry THF (0.49 mL) was added dropwise and the reaction mixture was stirred for 2 h 20 min until the solution became clear and yellow. After the addition of Na,K phosphate buffer (4 mL) the reaction mixture was extracted with EtOAc (3 × 8 mL) and the solvent evaporated. The crude product was purified with preparative HPLC (280 nm, A:B 10:90, 10 mL/min, t_R = 8.5 min) to give *N*-benzyl-5-hydroxy-3-(cyclohexanecarbonyl)tetramic acid (**12**, 14.0 mg, 44 µmol, 27% yield) as a white solid.

N-Benzyl-5-hydroxy-3-(cyclohexanecarbonyl)tetramic acid (12)

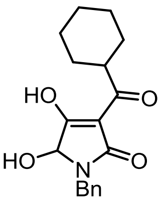

^{1}H NMR (500 MHz, $CDCl_3$): δ [ppm] = 7.35-7.26 (m, 5H), 5.02 (d, *J* = 14.80 Hz, 1H), 4.81 (s, 1H), 4.26 (d, *J* = 14.80 Hz, 1H), 3.30 (tt, *J* = 3.25, 11.72 Hz, 1H), 1.84-1.68 (m, 5H), 1.57-1.45 (m, 2H), 1.40-1.29 (m, 2H), 1.27-1.17 (m, 1H).

^{13}C NMR (125 MHz, $CDCl_3$): δ [ppm] = 192.8, 192.6, 173.1, 135.5, 128.9, 128.7, 128.0, 98.1, 81.0, 42.6, 41.2, 28.8, 28.6, 25.6, 25.5, 25.5.

HR-ES-MS for $[M-H]^-$ $C_{18}H_{20}NO_4$: calc. 314.1392, found 314.1388.

N-Benzyl-5-((benzenesulfonamide)(phenyl)methyl)-3-(cyclohexanecarbonyl)tetramic acid (15)

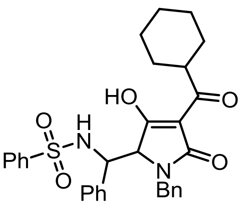

By using racemic Davis reagent II (**14**) instead of Davis reagent I (**13**) the title compound was isolated as a byproduct in 15% yield.

^{1}H NMR (500 MHz, $CDCl_3$): δ [ppm] = 7.65 (d, *J* = 7.45 Hz, 2H), 7.47 (t, *J* = 7.48 Hz, 1H), 7.38-7.27 (m, 5H), 7.20-7.11 (m, 5H), 6.94 (d, *J* = 7.05 Hz, 2H), 5.50 (d, *J* = 6.24 Hz, 1H), 5.16 (d, *J* = 15.11 Hz, 1H), 4.72 (dd, *J* = 4.14, 6.24 Hz, 1H), 3.94 (d, *J* = 3.90 Hz, 1H), 3.87 (d, *J* = 15.11 Hz, 1H), 3.22 (tt, *J* = 3.16, 16.96 Hz, 1H), 1.77 (t, *J* = 13.50 Hz, 2H), 1.69 (d, *J* = 12.51 Hz, 2H), 1.60 (d, *J* = 12.97 Hz, 1H), 1.50-1.14 (m, 5H).

^{13}C NMR (125 MHz, $CDCl_3$): δ [ppm] = 193.2, 191.6, 174.6, 139.7, 135.8, 135.1, 132.8, 129.1, 129.0, 128.6, 128.4, 128.2, 127.4, 127.0, 99.6, 66.7, 58.0, 44.2, 41.0, 28.6, 28.5, 25.6, 25.5, 25.4.

HR-ESI-MS for $[M+NH_4]^+$ $C_{31}H_{36}N_3O_5S$: calc. 562.2370, found 562.2378.

Oxidation with *t*-BuOOBz

Under an argon atmosphere, methyl lithium (1.19 M in Et_2O, 0.28 mL, 0.33 mmol, 2.5 equiv) was added dropwise at −78 °C to a solution of diisopropyl amine (47 µL, 0.33 mmol, 2.5 equiv) in dry THF (0.53 mL) and stirred for 1.5 h at −78 °C. Then a solution of *N*-benzyl-tetramic acid **7** (40 mg, 0.13 mmol, 1.0 eq) in dry THF (0.27 mL) was added and the cloudy orange solution was stirred for 20 min. Thereafter a solution of *tert*-butyl peroxybenzoate (38.1 µL, 0.20 mmol, 1.5 equiv) in dry THF (0.39 mL) was added over 4 min at −78 °C. After stirring for 1.5 h, the clear and light yellow reaction mixture was quenched with Na,K phosphate buffer (2 mL) and extracted with EtOAc (3 × 7 mL). Evaporation of the solvent under reduced pressure delivered an orange oil which was further purified by HPLC (210 nm, A:B 40:60 (8 mL/min) → 40:40 (8 mL/min, 5. min) → 0:100 (10 mL/min, 30. min) → 0:100 (10 mL/min, 35. min)). As main compound **S1** (t_R = 31.7 min) was isolated as a light red solid (4.5 mg, 11 µmol, 8% yield) along with 1.7 mg of the starting tetramic acid (t_R = 26.9 min).

N-Benzyl-5-benzoyl-3-(cyclohexancarbonyl)tetramic acid (S1)

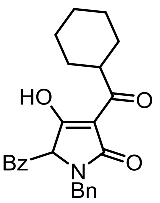

^{1}H NMR (500 MHz, $CDCl_3$): δ [ppm] = 7.96 (d, *J* = 7.30 Hz, 2H), 7.61 (t, *J* = 7.43 Hz, 1H), 7.48 (t, *J* = 7.83 Hz, 2H), 7.29-7.25 (m, 3H), 7.16-7.12 (m, 2H), 5.40 (d, *J* = 14.88 Hz, 1H), 5.28 (s, 1H), 3.95 (d, *J* = 14.88 Hz, 1H), 3.25 (tt, J = 2.97, 17.69 Hz, 1H), 1.81-1.61 (m, 5H), 1.54-1.44 (m, 2H), 1.36-1.13 (m, 3H).

^{13}C NMR (125 MHz, $CDCl_3$): δ [ppm] = 193.6, 190.0, 185.7, 174.3, 135.0, 134.9, 134.3, 129.9, 129.1, 128.7, 128.7, 128.2, 98.6, 69.9, 44.1, 41.1, 29.0, 28.3, 25.6, 25.4.

HR-ESI-MS for $[M+H]^+$ $C_{25}H_{26}NO_4$: calc. 404.1856, found 404.1870.

Oxidation with molecular oxygen

Under an argon atmosphere, KHMDS (1 M in THF, 2.5 equiv) was added at −78 °C to a solution of N-protected 3-(cyclohexancarbonyl)tetramic acid (1.0 equiv) in dry THF (0.2 M) and stirred for 25 min. After addition of $P(OEt)_3$ (2.0 equiv), oxygen (predried over silica and P_4O_{10}, both with moisture indicator) was passed through the bright yellow reaction mixture until full conversion (change of color from bright to light yellow and TLC control). Saturated NH_4Cl solution (3 mL) was added and the reaction mixture was extracted with EtOAc. After evaporation of the solvent the residue was extracted with SPE (H_2O, then MeCN) and the organic phase was purified with preparative HPLC to give the corresponding 5-hydroxy-3-(cyclohexanecarbonyl)tetramic acid as a white solid.

***N*-(2,4-Dimethoxybenzyl)-5-hydroxy-3-(cyclohexanecarbonyl)tetramic acid (16)**

On a 0.72 mmol scale the title compound was isolated as a white solid (167.5 mg, 0.45 mmol, 62% yield, 69% brsm). Further 27.6 mg of the starting material was recovered.

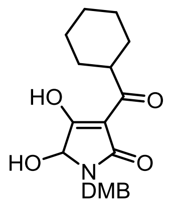

t_R = 8.2 min (280 nm, 10:90 A:B, 10 mL/min).

^{1}H NMR (500 MHz, $CDCl_3$): δ [ppm] = 7.24-7.21 (m, 1H), 6.46-6.42 (m, 2H), 4.85 (s, 1H), 4.78 (d, *J* = 14.50 Hz, 1H), 4.33 (d, *J* = 14.50 Hz, 1H), 3.81 (s, 3H), 3.77 (s, 3H), 3.30 (tt, *J* = 3.13, 11.63 Hz, 1H), 1.80-1.64 (m, 5H), 1.53-1.42 (m, 2H), 1.38-1.27 (m, 2H), 1.24-1.14 (m, 1H).

^{13}C NMR (125 MHz, $CDCl_3$): δ [ppm] = 192.1, 191.8, 173.1, 160.9, 158.5, 131.8, 116.1, 104.6, 98.7, 98.2, 81.4, 55.6, 55.4, 40.9, 37.2, 28.7, 28.5, 25.5, 25.4, 25.4.

HR-ESI-MS for $[M+H]^+$ $C_{20}H_{26}NO_6$: calc. 376.1755, found 376.1759; for $[M+Na]^+$ $C_{20}H_{25}NNaO_6$: calc. 398.1574, found 398.1577.

***N*-Allyl-5-hydroxy-3-(cyclohexanecarbonyl)tetramic acid (17)**

On a 0.17 mmol scale the title compound was isolated as a white solid (18.6 mg, 70 µmol, 42% yield, 44% brsm) after stirring for 4 h during which period the cooling bath was allowed to warm up from −78 °C to room temperature.

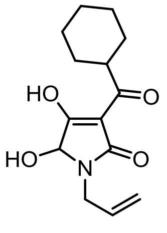

t_R = 7.9 min (280 nm, 15:85 A:B, 9 mL/min).

Mixture of tautomers 1:0.2.

^{1}H NMR (500 MHz, $CDCl_3$): δ [ppm] = 5.75 (dddd, *J* = 4.91, 7.25, 10.05, 17.20 Hz, 1.2H), 5.26-5.14 (m, 2.4H), 5.06 (s, 0.2H, minor tautomer) 4.90 (s, 1H), 4.28 (tdd, *J* = 1.42, 4.82, 15.42 Hz, 1.2H), 3.78 (dd, *J* = 7.35, 15.46 Hz, 1.2H), 3.26 (tt, *J* = 3.40, 17.35 Hz, 1.2H), 1.84-1.57 (m, 11H), 1.52-1.40 (m, 4H), 1.40-1.06 (m, 13H).

^{13}C NMR (175 MHz, $CDCl_3$): δ [ppm] = 25.4, 25.4, 25.5, 28.6, 28.7, 41.0, 41.5, 81.4, 97.9, 119.0, 131.6, 173.2, 191.8, 192.4.

HR-ESI-MS for $[M+H]^+$ $C_{14}H_{20}NO_4$: calc. 266.1387, found 266.1380. HR-ESI-MS for $[M+Na]^+$ $C_{14}H_{19}NNaO_4$: calc. 288.1206, found 288.1202.

Ether syntheses

N-(2,4-Dimethoxybenzyl)-5-methoxy-3-(cyclohexanecarbonyl)tetramic acid (18)

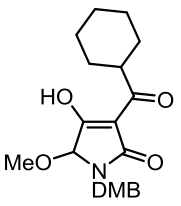

Tetramic acid **17** (15.9 mg, 42 µmol, 1.0 equiv) was dissolved in methanol (5 mL) and heated to 60 °C for 1 h. Evaporation gave the title compound as a colorless resin (15.7 mg, 40 µmol, 95% yield).

^1H NMR (500 MHz, $CDCl_3$): δ [ppm] = 7.16 (d, *J* = 8.96 Hz, 1H), 6.41-6.36 (m, 2H), 4.77 (d, *J* = 14.51 Hz, 1H), 4.58 (s, 1H), 4.16 (d, *J* = 14.51 Hz, 1H), 3.74 (s, 3H), 3.73 (s, 3H), 3.28 (s, 3H), 1.77-1.61 (m, 5H), 1.46 (tq, *J* = 3.43, 20.64 Hz, 2H), 1.36-1.24 (m, 2H), 1.22-1.10 (m, 1H).

^{13}C NMR (125 MHz, $CDCl_3$): δ [ppm] = 191.3, 191.3, 173.7, 160.9, 158.6, 131.7, 116.2, 104.2, 99.0, 98.6, 87.4, 55.4, 55.4, 53.8, 40.9, 37.6, 28.8, 28.6, 25.6, 25.5, 25.5.

HR-ESI-MS for $[M+H]^+$ $C_{21}H_{28}NO_6$: calc. 390.1911, found 390.1910. HR-ESI-MS for $[M+Na]^+$ $C_{21}H_{27}NNaO_6$: calc. 412.1731, found 412.1731.

N-(2,4-Dimethoxybenzyl)-5-ethoxy-3-(cyclohexanecarbonyl)tetramic acid (19)

Tetramic acid **17** (10.3 mg, 27 µmol, 1.0 equiv) was dissolved in ethanol (5 mL) and heated to 60 °C for 1 h. Evaporation of the solvent gave the title compound as a colorless resin (10.2 mg, 25 µmol, 92% yield).

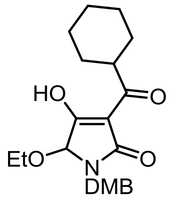

^1H NMR (500 MHz, $CDCl_3$): δ [ppm] = 7.15 (d, *J* = 9.00 Hz, 1H), 6.42-6.36 (m, 2H), 4.75 (d, *J* = 14.71 Hz, 1H), 4.60 (s, 1H), 4.19 (d, *J* = 14.71 Hz, 1H), 3.73 (s, 3H), 3.73 (s, 3H), 3.64 (qd, *J* = 7.15, 8.37 Hz, 1H), 3.42 (qd, *J* = 7.15, 8.37 Hz, 1H), 3.27 (tt, *J* = 3.21, 11.73 Hz, 1H), 1.76-1.60 (m, 5H), 1.50-1.38 (m, 2H), 1.37-1.17 (m, 3H), 1.15 (t, *J* = 7.15 Hz, 3H).

^{13}C NMR (125 MHz, $CDCl_3$): δ [ppm] = 191.6, 191.1, 173.5, 160.9, 158.6, 131.6, 116.2, 104.2, 98.9, 98.6, 87.0, 62.8, 55.4, 40.9, 37.5, 28.7, 28.6, 25.6, 25.5, 25.5, 15.2.

HR-ESI-MS for $[M+H]^+$ $C_{22}H_{30}NO_6$: calc. 404.2068, found 404.2062. HR-ESI-MS for $[M+Na]^+$ $C_{22}H_{29}NNaO_6$: calc.426.1887, found 426.1882.

N-(2,4-Dimethoxybenzyl)-5-(2-(trimethylsilyl)ethoxy)-3-(cyclohexanecarbonyl)tetramic acid (20)

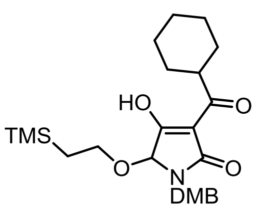

Tetramic acid **17** (10.4 mg, 28 µmol, 1.0 equiv) was dissolved in 2-(trimethylsilyl)ethanol (60 µL, 49.8 mg, 421 µmol, 15.0 equiv) and the solution was heated to 60 °C for 1 h. Evaporation of the solvent gave the title compound as a colorless resin (12.2 mg, 26 µmol, 92% yield).

^{1}H NMR (500 MHz, $CDCl_3$): δ [ppm] = 7.34-7.19 (m, 1H), 6.47-6.42 (m, 2H), 4.80 (d, *J* = 14.5 Hz, 1H), 4.63 (s, 1H), 4.26 (d, *J* = 14.5 Hz, 1H), 3.79 (s br, 6H), 3.78-3.71 (m, 1H), 3.53-3.45 (m, 1H), 3.33 (tt, *J* = 3.2, 11.7 Hz, 1H), 1.82-1.66 (m, 5H), 1.55-1.44 (m, 2H), 1.42-1.29 (m, 2H), 1.27-1.15 (m, 2H), 0.98-0.90 (m, 1H), 0.00 (s, 9H).

^{13}C NMR (125 MHz, $CDCl_3$): δ [ppm] = 193.2, 192.4, 174.9, 162.2, 160.0, 133.0, 117.7, 105.5, 100.2, 99.9, 88.3, 66.7, 56.8, 42.2, 38.9, 30.1, 30.0, 27.0, 26.9, 19.8, 0.0.

HR-ESI-MS for $[M+H]^+$ $C_{25}H_{38}NO_6Si$: calc. 476.2463, found 476.2456. HR-ESI-MS for $[M+Na]^+$ $C_{25}H_{37}NNaO_6Si$: calc. 498.2282, found 498.2277.

Attempted benzyl deprotection with PtO_2

Under an argon atmosphere, platinum(IV) oxide (16.0 mg, 70 µmol, 0.98 equiv) was added to a solution of 5-hydroxylated tetramic acid **12** (22.6 mg, 72 µmol, 1.0 equiv) in dry $MeOH/CH_2Cl_2$ (1:1 v/v, 1.44 mL). Then hydrogen from a balloon was bubbled through the stirred reaction mixture for 10 min and stirring under hydrogen was continued for additional 25 min. After filtration through Celite washing with CH_2Cl_2 and evaporation of the solvent, the crude product mixture was purified with preparative HPLC (A:B 50:50 (8 mL/min) → 50:50 (8 mL/min, 5. min) → 0:100 (10 mL/min, 35. min) → 0:100 (10 mL/min, 45. min)). The main compound **S2** (t_R = 38.1 min) was isolated as a colorless solid (2.9 mg, 10.4 µmol, 15% yield).

1,3-Bis(cyclohexylmethyl)pyrrolidin-2-one (S2)

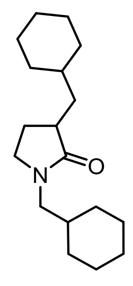

^{1}H NMR (700 MHz, $CDCl_3$): δ [ppm] = 3.30-3.22 (m, 2H), 3.11 (dd, *J* = 7.10, 13.55 Hz, 1H), 3.05 (dd, *J* = 7.11, 13.59 Hz, 1H), 2.46 (tdd, *J* = 8.64, 4.12, 10.76 Hz, 1H), 2.17 (dddd, *J* = 3.90, 6.76, 12.36, 8.67 Hz, 1H), 1.78 (ddd, *J* = 4.30, 9.25, 13.66 Hz, 1H), 1.75-1.53 (m, 8H), 1.37-1.30 (m, 1H), 1.27-1.10 (m, 7H), 1.00-0.83 (m, 4H).

^{13}C NMR (175 MHz, $CDCl_3$): δ [ppm] = 177.5, 49.1, 46.2, 39.6, 39.3, 36.0, 35.6, 34.3, 32.2, 30.8, 26.6, 26.5, 26.4, 26.3, 25.9, 25.9.

HR-ESI-MS for $[M+Na]^+$ $C_{18}H_{31}NNaO$: calc. 300.2298, found 300.2296.

Attempted benzyl deprotection with Raney–Ni

Under an argon atmosphere, Raney–Ni (freshly prepared, small spatula tip) was added to a solution of 5-hydroxylated tetramic acid **12** (15.4 mg, 49 µmol, 1.0 equiv) in dry ethanol (1.5 mL). A hydrogen-filled balloon was attached and the reaction mixture was stirred for 5.5 h. Filtration through Celite, washing with ethanol and evaporation of the solvent gave a white solid which was further purified with preparative HPLC (A:B 50:50 (8 mL/min) → 50:50 (8 mL/min, 5. min) → 0:100 (10 mL/min, 35. min) → 0:100 (10 mL/min, 45. min) to give the starting material (4.4 mg, t_R = 25.2 min) and the corresponding ethyl ether **S3** (t_R = 36.1 min, 1.5 mg, 4 µmol, 9% yield).

***N*-Benzyl-5-ethoxy-3-(cyclohexancarbonyl)tetramic acid (S3)**

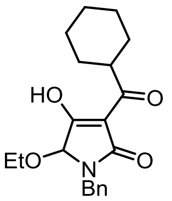

^{1}H NMR (500 MHz, $CDCl_3$): δ [ppm] = 7.37-7.26 (m, 5H), 4.98 (d, *J* = 14.59 Hz, 1H), 4.60 (s, 1H), 4.17 (d, *J* = 14.59 Hz, 1H), 3.60 (q, *J* = 7.02 Hz, 1H), 3.45 (q, *J* = 7.02 Hz, 1H), 3.35 (tt, *J* = 3.28, 11.72 Hz, 1H), 1.83-1.67 (m, 5H), 1.56-1.46 (m, 2H), 1.43-1.29 (m, 2H), 1.27-1.19 (m, 1H), 1.17 (t, *J* = 7.02 Hz, 3H).

^{13}C NMR (125 MHz, $CDCl_3$): δ [ppm] = 191.7, 191.0, 173.4, 135.6, 128.9, 128.7, 128.0, 98.9, 86.4, 62.5, 42.7, 40.9, 28.8, 28.6, 25.6, 25.5, 25.5, 15.1.

HR-ESI-MS for $[M+Na]^+$ $C_{20}H_{25}NNaO_4$: calc. 366.1676, found 366.1677.

DMB-deprotection with DDQ

5-(2-(Trimethylsilyl)ethoxy)-3-(cyclohexanecarbonyl)tetramic acid (21)

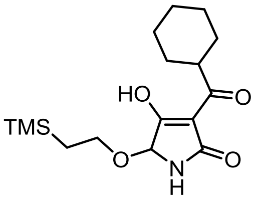

DDQ (20.4 mg, 90 µmol, 3.5 equiv) was added in one portion at room temperature to a vigorously stirred solution of (trimethylsilyl)ethyl hemiaminal ether **20** (12.2 mg, 26 µmol, 1.0 equiv) in H_2O/CH_2Cl_2 (1:19, 2 mL) to give a light green solution. After stirring for 24 h at room temperature, CH_2Cl_2 (3 mL) was added and the solvent was removed under reduced pressure. Purification with HPLC (280 nm, A:B 10:90, 9 mL/min) gave the N-deprotected tetramic acid **21** (t_R = 3.4 min, 2.3 mg, 7.07 µmol, 28% yield, 45% brsm) as a red resin. In addition 4.8 mg of the starting material were recovered (t_R = 8.6 min).

Mixture of tautomers 1:0.8.

^{1}H NMR (700 MHz, CD_3CN): δ [ppm] = 7.33 (s, 1H), 7.19 (s, 1H), 4.94 (s, 0.8H), 4.84 (d, *J* = 1.54 Hz, 1H), 3.67 (dt, *J* = 6.47, 14.34 Hz, 1.5H), 3.62 (dt, *J* = 6.40, 14.66 Hz, 1.5H), 3.29 (qt, *J* = 3.56, 17.82 Hz, 2H), 1.83-1.78 (m, 6H), 1.78-1.72 (m, 5.4H), 1.72-1.67 (m, 4H), 1.52-1.44 (m, 5.7H), 1.39-1.30 (m, 7H), 1.30-1.21 (m, 5.5H), 0.92 (ddd, *J* = 6.83, 7.58, 9.63 Hz, 4H), 0.01 (s, 11H).

^{13}C NMR (125 MHz, CD_3CN): δ [ppm] = 193.5, 192.9, 192.6, 192.0, 177.1, 172.6, 151.5, 99.3, 85.7, 80.5, 66.3, 41.8, 41.8, 29.4, 29.4, 29.3, 29.3, 26.3, 18.7, -1.4.

HR-ESI-MS for $[M+H]^+$ $C_{16}H_{28}NO_4Si$: calc. 326.1782, found 326.1778. HR-ESI-MS for $[M+Na]^+$ $C_{16}H_{27}NNaO_4Si$: calc. 348.1602, found 348.1599.

5-Hydroxy-3-(cyclohexanecarbonyl)tetramic acid (22)

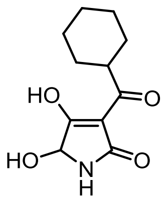

DDQ (31 mg, 137 µmol, 3.6 equiv) was added in one portion at room temperature to a vigorously stirred solution of 5-hydroxytetramic acid **16** (14.6 mg, 38 µmol, 1.0 equiv) in H_2O/CH_2Cl_2 (1:19, 2.91 mL) to give a dark green solution. After stirring for 2.5 h and 7.5 h at room temperature additional portions of DDQ (each 33 mg, 145 µmol, 3.83 equiv) were added. After stirring for 21.5 h in total, CH_2Cl_2 (3 mL) was added to the now red solution and the solvent was removed under reduced pressure. Purification with HPLC (210 nm, A:B 50:50, 10 mL/min) gave the N-deprotected tetramic acid **22** (t_R = 9.1 min, 4.0 mg, 17.8 µmol, 47% yield, 77% brsm) as a pale orange resin. Additionally 5.6 mg of the starting tetramic acid were recovered (t_R = 32.7 min).

^1H NMR (700 MHz, CD_3CN): δ [ppm] = 7.14 (s, 1H), 4.94 (s, 1H), 4.44 (s, 1H), 3.31 (tt, J = 3.27, 17.94 Hz, 1.3H), 1.83-1.78 (m, 3.6H), 1.78-1.73 (m, 3H), 1.73-1.68 (m, 2H), 1.49 (dq, J = 3.47, 12.55 Hz, 3H), 1.39-1.31 (m, 3.4H), 1.25 (tq, J = 3.52, 12.82 Hz, 2H).

^{13}C NMR (175 MHz, CD_3CN): δ [ppm] = 193.4, 192.5, 176.7, 99.0, 80.4, 41.7, 29.4, 29.3, 26.3, 26.3, 26.3.

HR-ESI-MS for $[M+H]^+$ $C_{11}H_{16}NO_4$: calc. 226.1074, found 226.1070. HR-ESI-MS for $[M-H_2O+H]^+$ $C_{11}H_{14}NO_3$: calc. 208.0968, found 208.0963.
