# Supplementary material for: A simple and efficient method for the preparation of 5-hydroxy-3-acyltetramic acids
Source: Beilstein J Org Chem. 2015 Mar 6;11:323–7. doi: 10.3762/bjoc.11.37 (PMC4362042; doi:10.3762/bjoc.11.37)
Supplement: File 2 — Copies of NMR spectra. [file Beilstein_J_Org_Chem-11-323-s002.pdf]

Supporting Information

for

A simple and efficient method for the preparation of 5-hydroxy-3-acyltetramic acids

Johanna Trenner[1], Evgeny V. Prusov*[1]

Address: [1]Department of Medicinal Chemistry, Helmholtz Centre for Infection Research, Inhoffenstr. 7, 38124 Braunschweig, Germany

Email: Evgeny V. Prusov - evgeny.prusov@helmholtz-hzi.de

* Corresponding author

Copies of NMR spectra

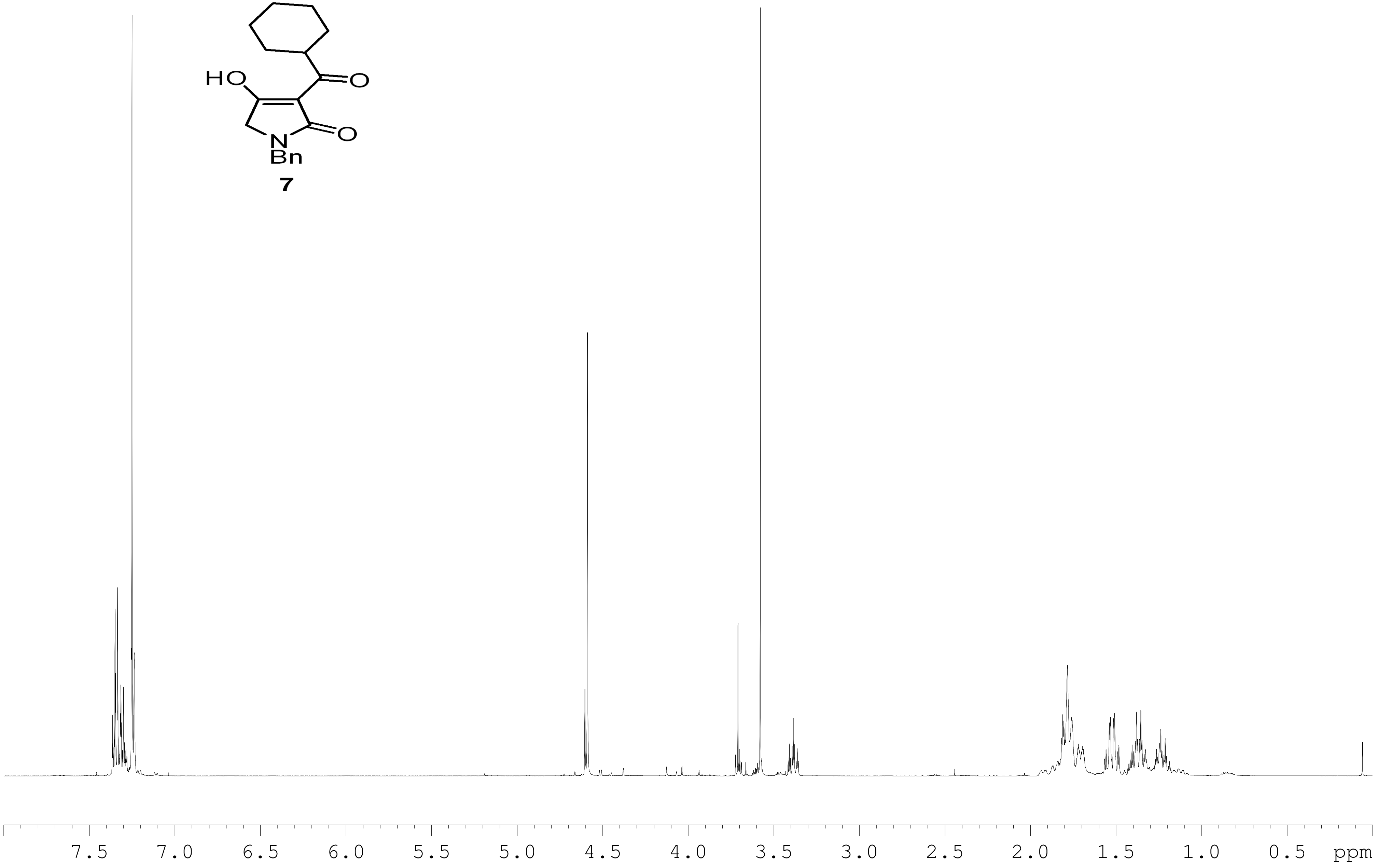
HO
O
O
N
Bn
7
7.5
7.0
6.5
6.0
5.5
5.0
4.5
4.0
3.5
3.0
2.5
2.0
1.5
1.0
0.5
ppm

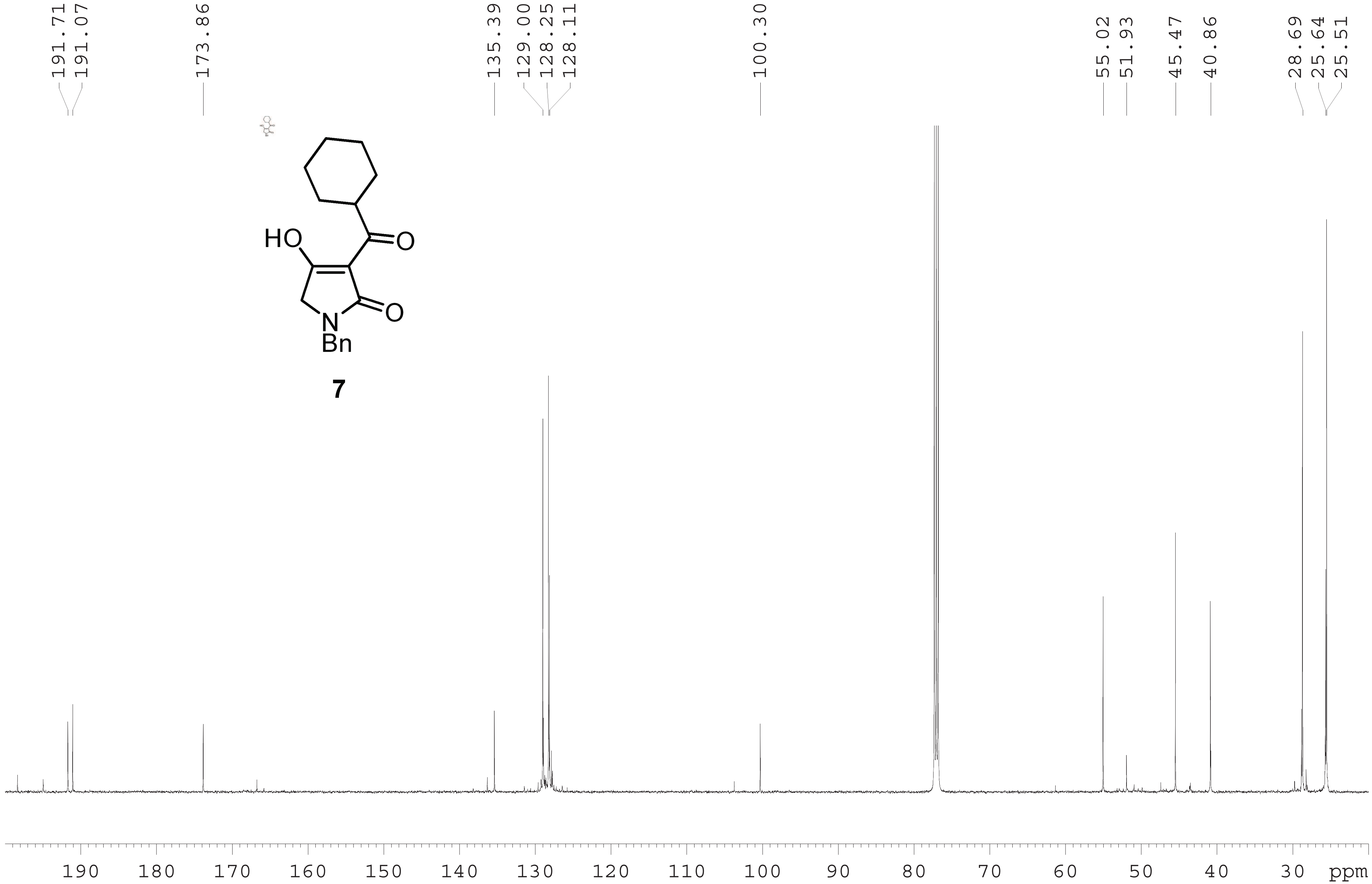
191.71
191.07
173.86
135.39
129.00
128.25
128.11
100.30
55.02
51.93
45.47
40.86
28.69
25.64
25.51
HO
O
O
N
Bn
7
190
180
170
160
150
140
130
120
110
100
90
80
70
60
50
40
30
ppm

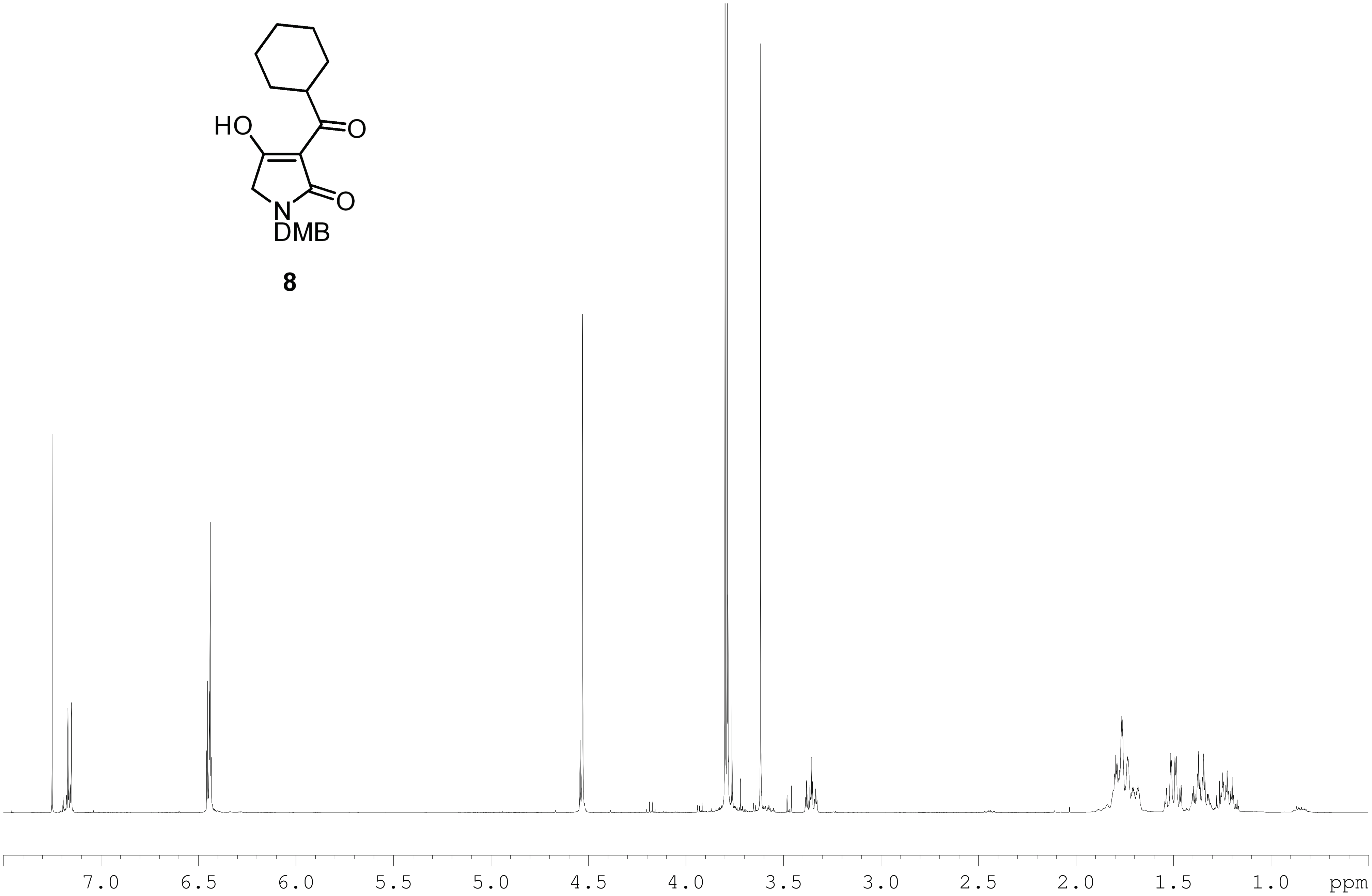
HO
O
O
N
DMB
8
7.0 6.5 6.0 5.5 5.0 4.5 4.0 3.5 3.0 2.5 2.0 1.5 1.0 ppm

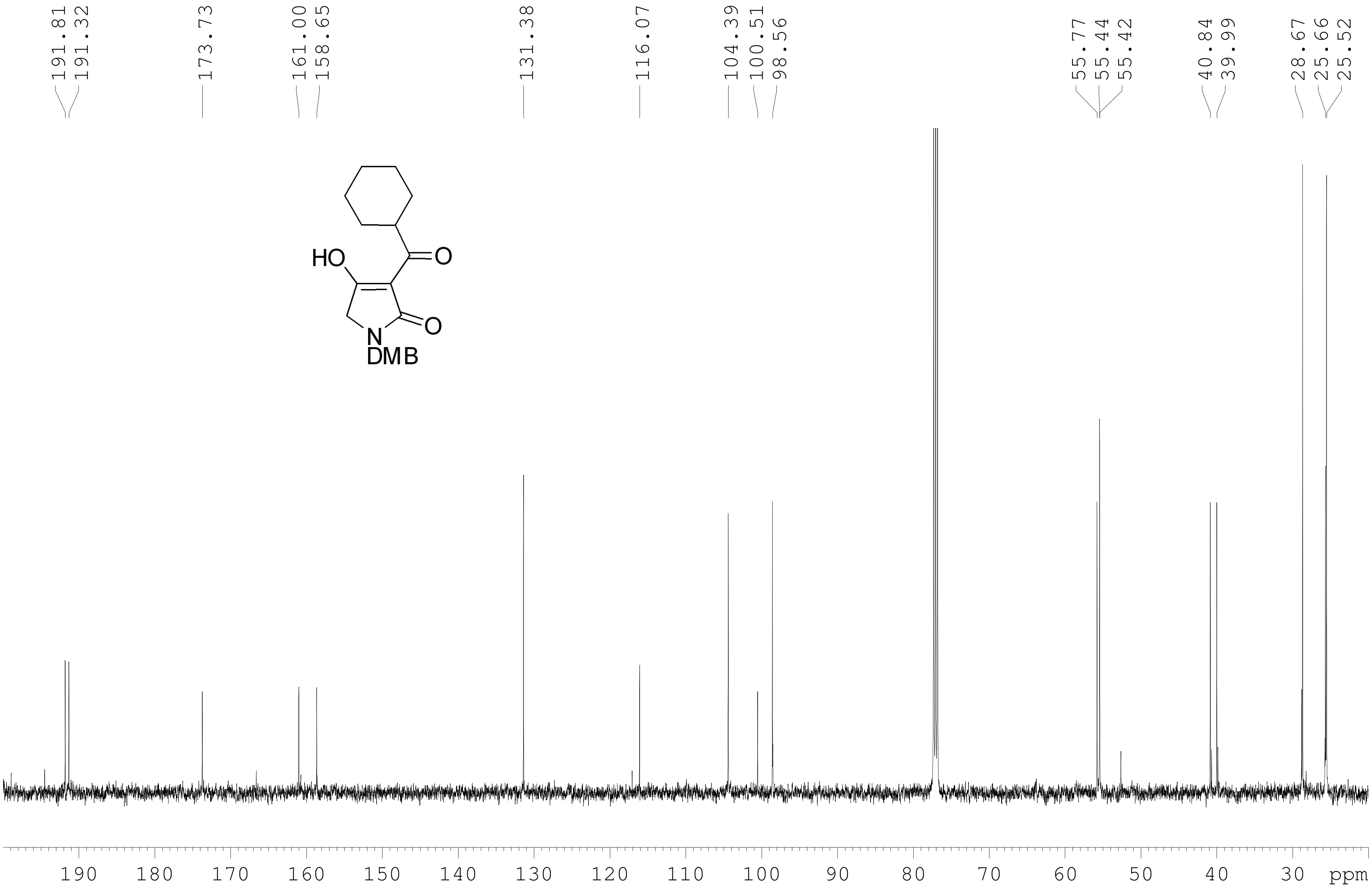
191.81
191.32
173.73
161.00
158.65
131.38
116.07
104.39
100.51
98.56
55.77
55.44
55.42
40.84
39.99
28.67
25.66
25.52
HO
O
O
N
DMB
190
180
170
160
150
140
130
120
110
100
90
80
70
60
50
40
30
ppm

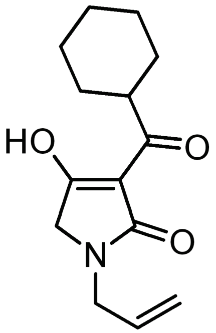

11

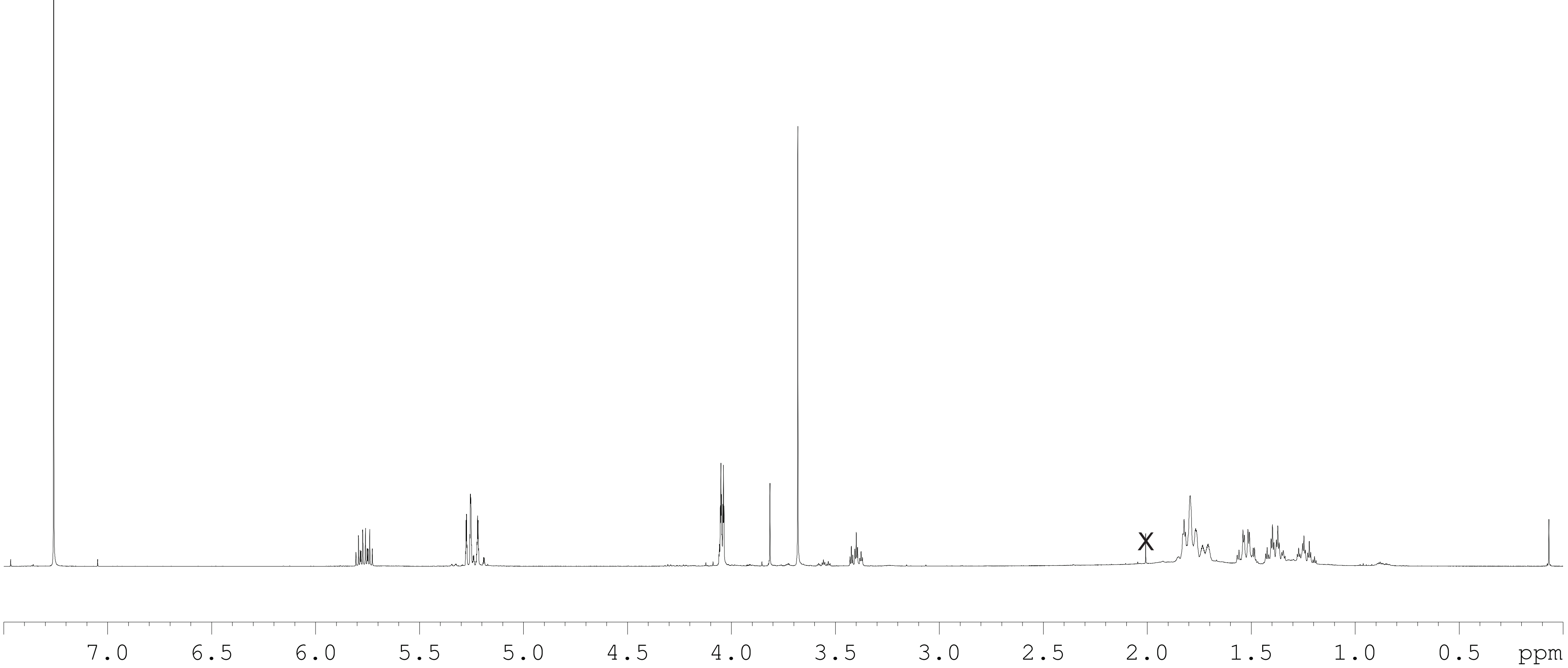

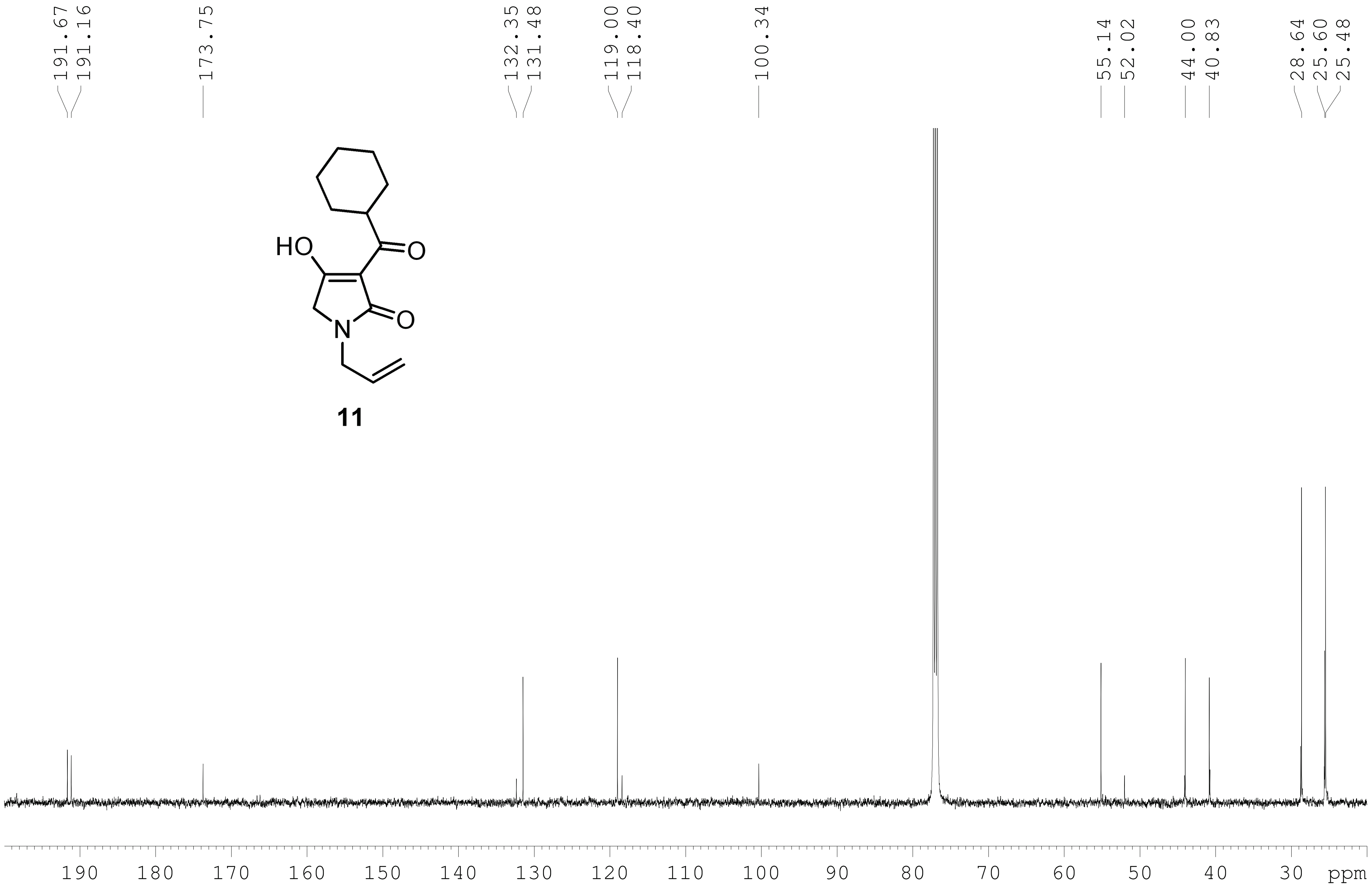
191.67
191.16
173.75
132.35
131.48
119.00
118.40
100.34
55.14
52.02
44.00
40.83
28.64
25.60
25.48
HO
O
N
O
11
190
180
170
160
150
140
130
120
110
100
90
80
70
60
50
40
30
ppm

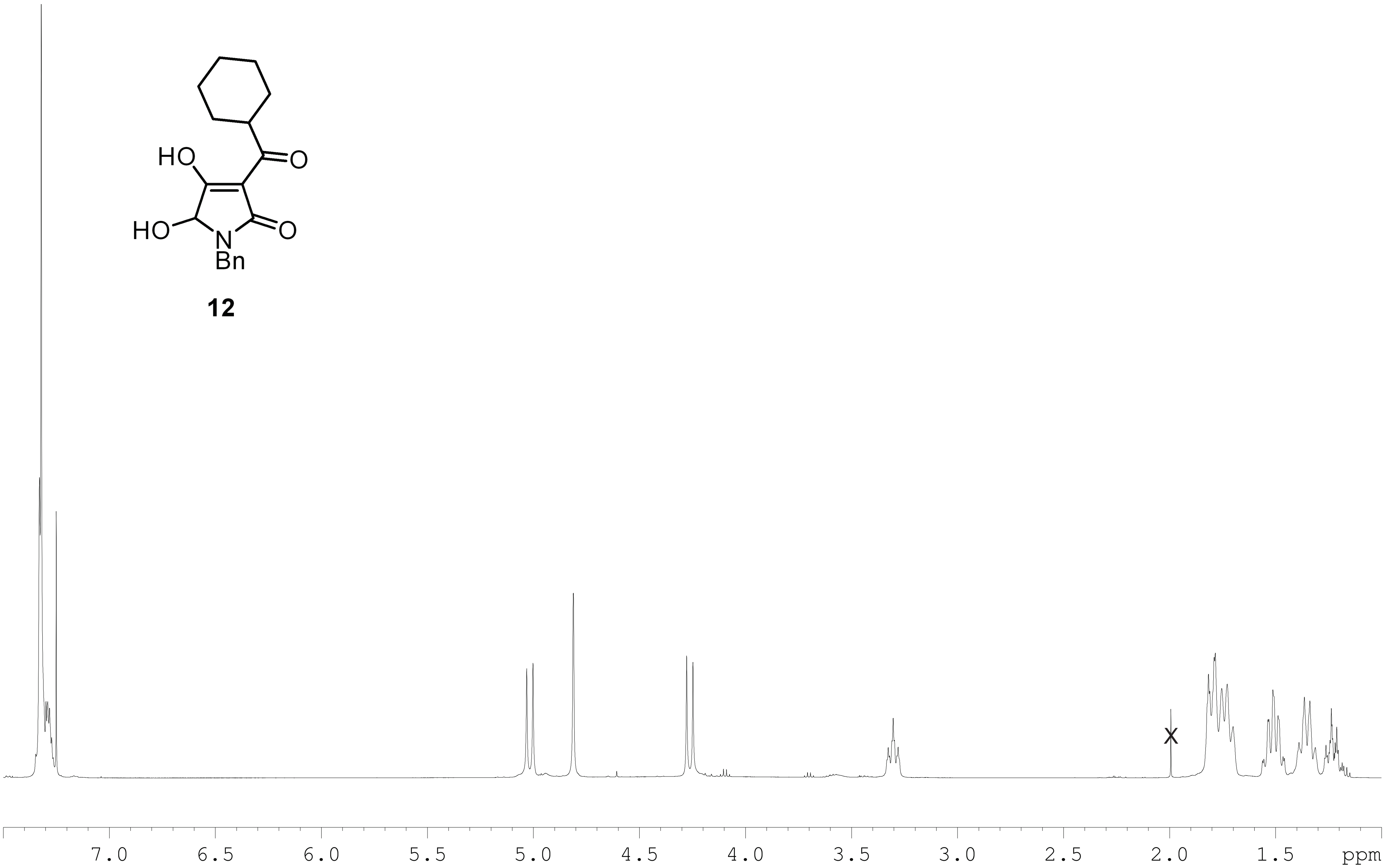

12

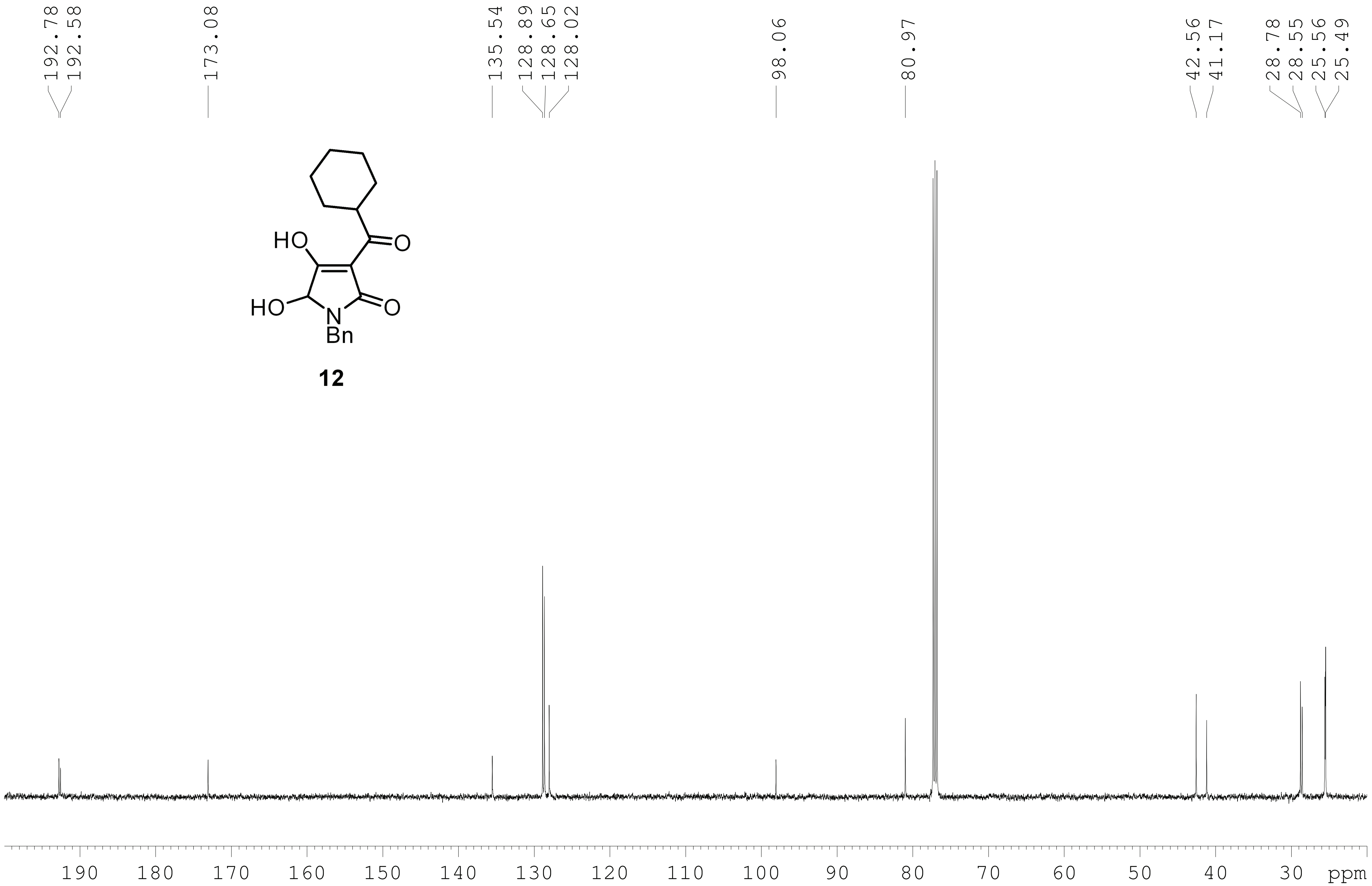
192.78
192.58
173.08
135.54
128.89
128.65
128.02
98.06
80.97
42.56
41.17
28.78
28.55
25.56
25.49
HO
O
HO
N
O
Bn
12
190 180 170 160 150 140 130 120 110 100 90 80 70 60 50 40 30 ppm

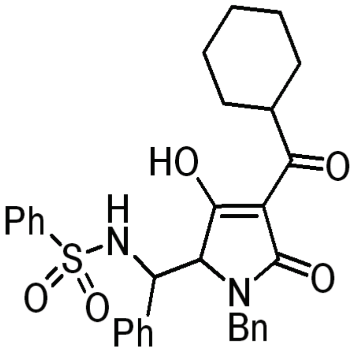

15

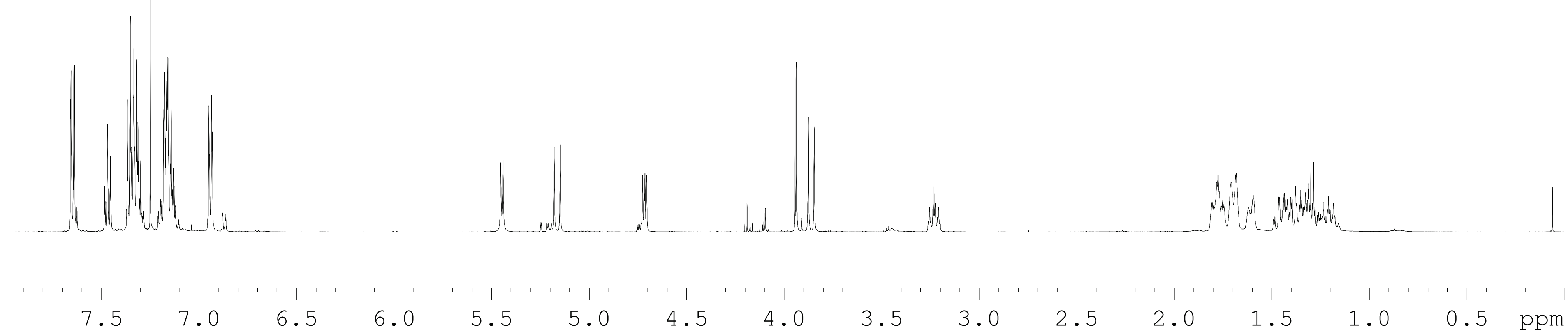

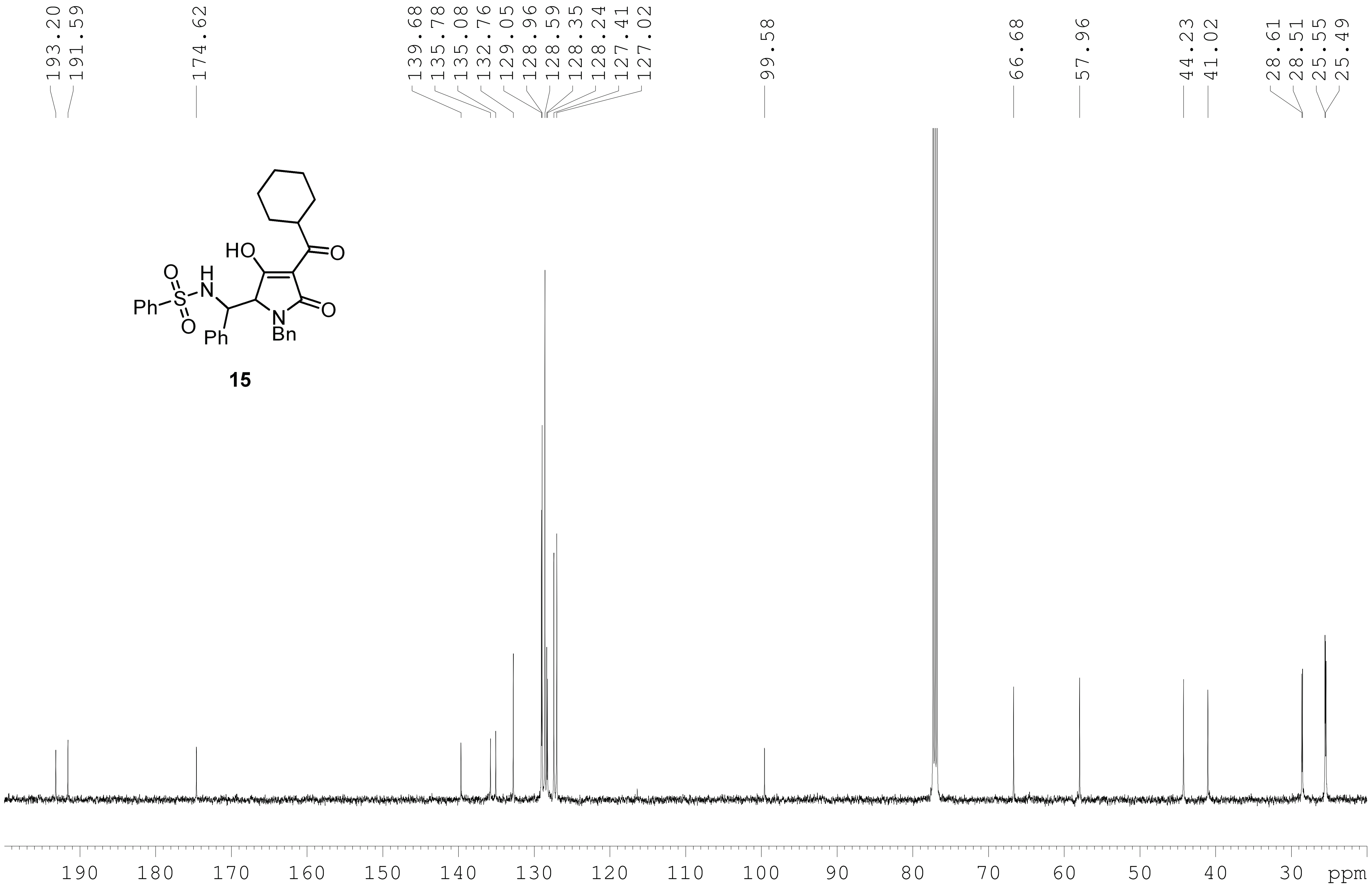
193.20
191.59
174.62
139.68
135.78
135.08
132.76
129.05
128.96
128.59
128.35
128.24
127.41
127.02
99.58
66.68
57.96
44.23
41.02
28.61
28.51
25.55
25.49
HO
O
H
N
Ph
S
O
Ph
N
Bn
O
15
190 180 170 160 150 140 130 120 110 100 90 80 70 60 50 40 30 ppm

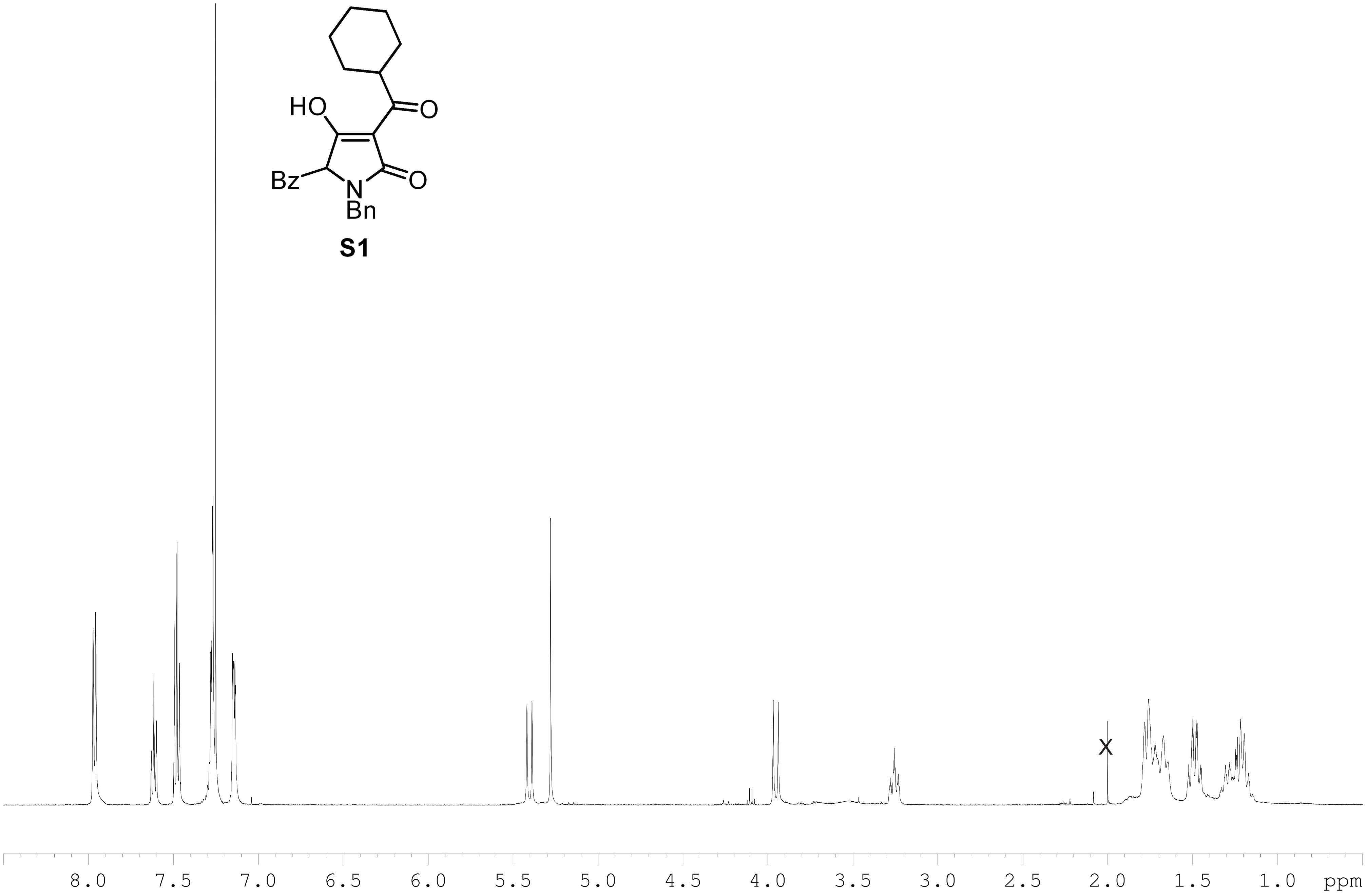
HO
O
Bz
N
O
Bn
S1
X
8.0 7.5 7.0 6.5 6.0 5.5 5.0 4.5 4.0 3.5 3.0 2.5 2.0 1.5 1.0 ppm

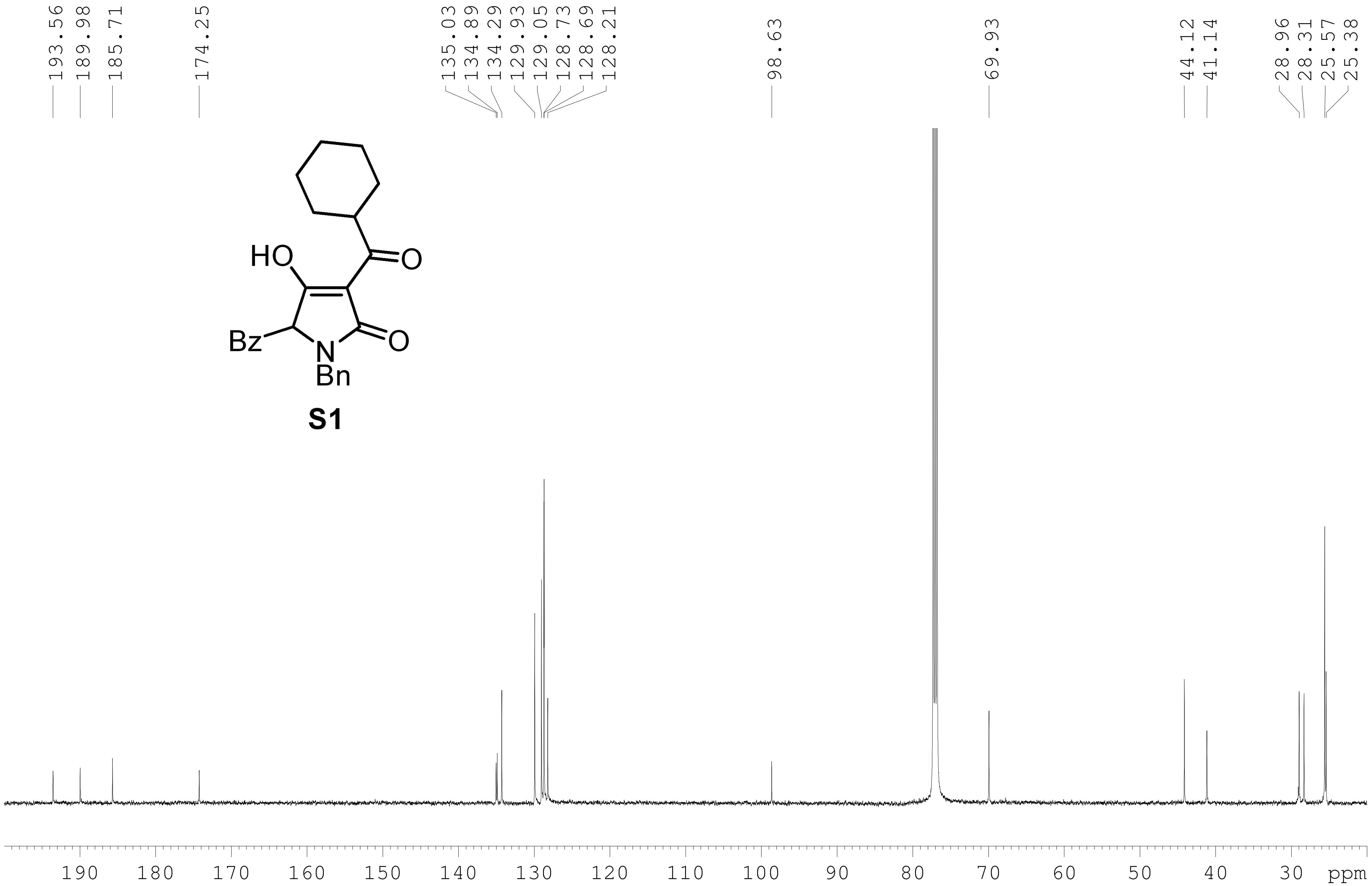
193.56
189.98
185.71
174.25
135.03
134.89
134.29
129.93
129.05
128.73
128.69
128.21
98.63
69.93
44.12
41.14
28.96
28.31
25.57
25.38
HO
O
Bz
N
Bn
O
S1
190
180
170
160
150
140
130
120
110
100
90
80
70
60
50
40
30
ppm

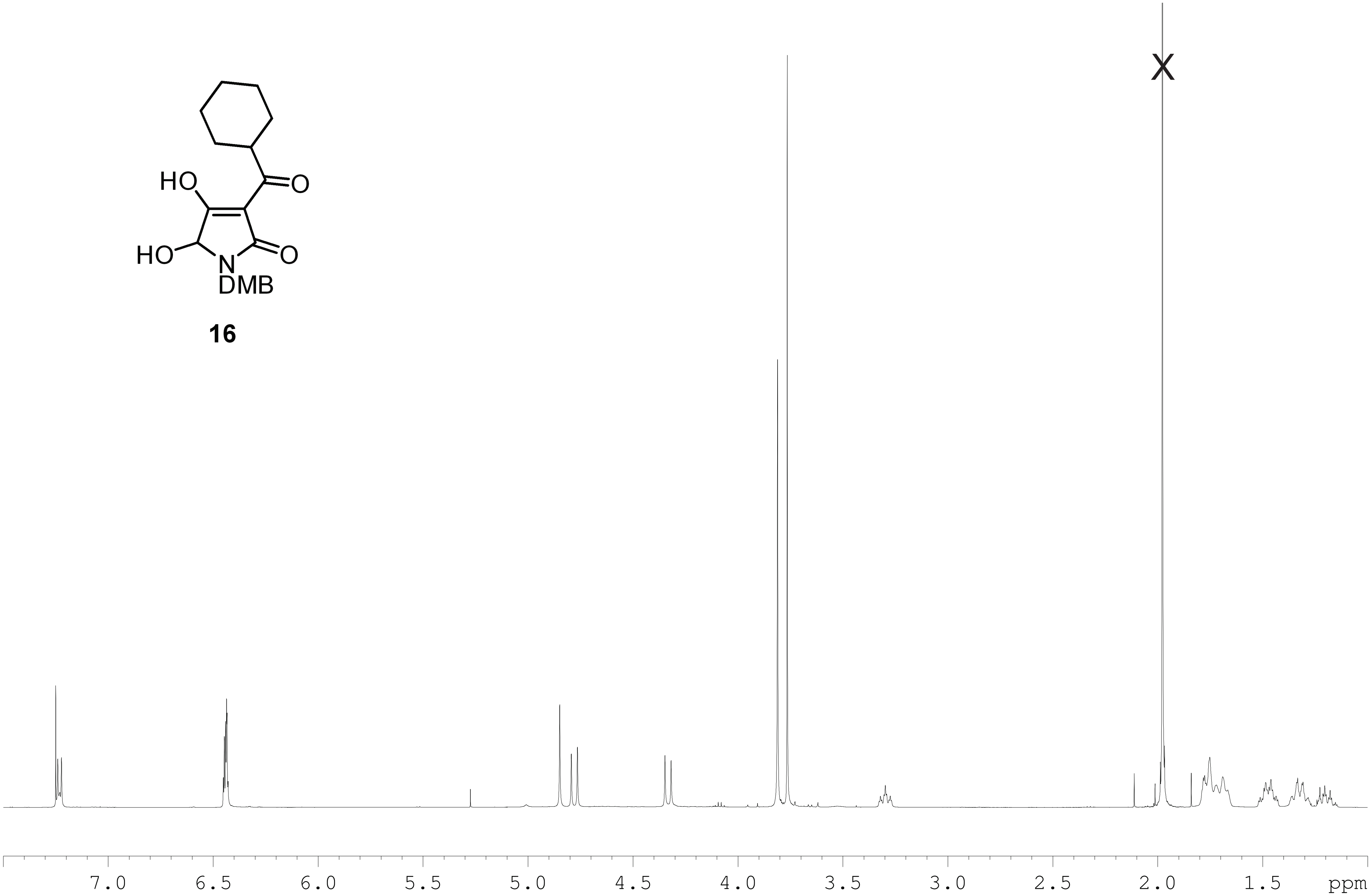

16

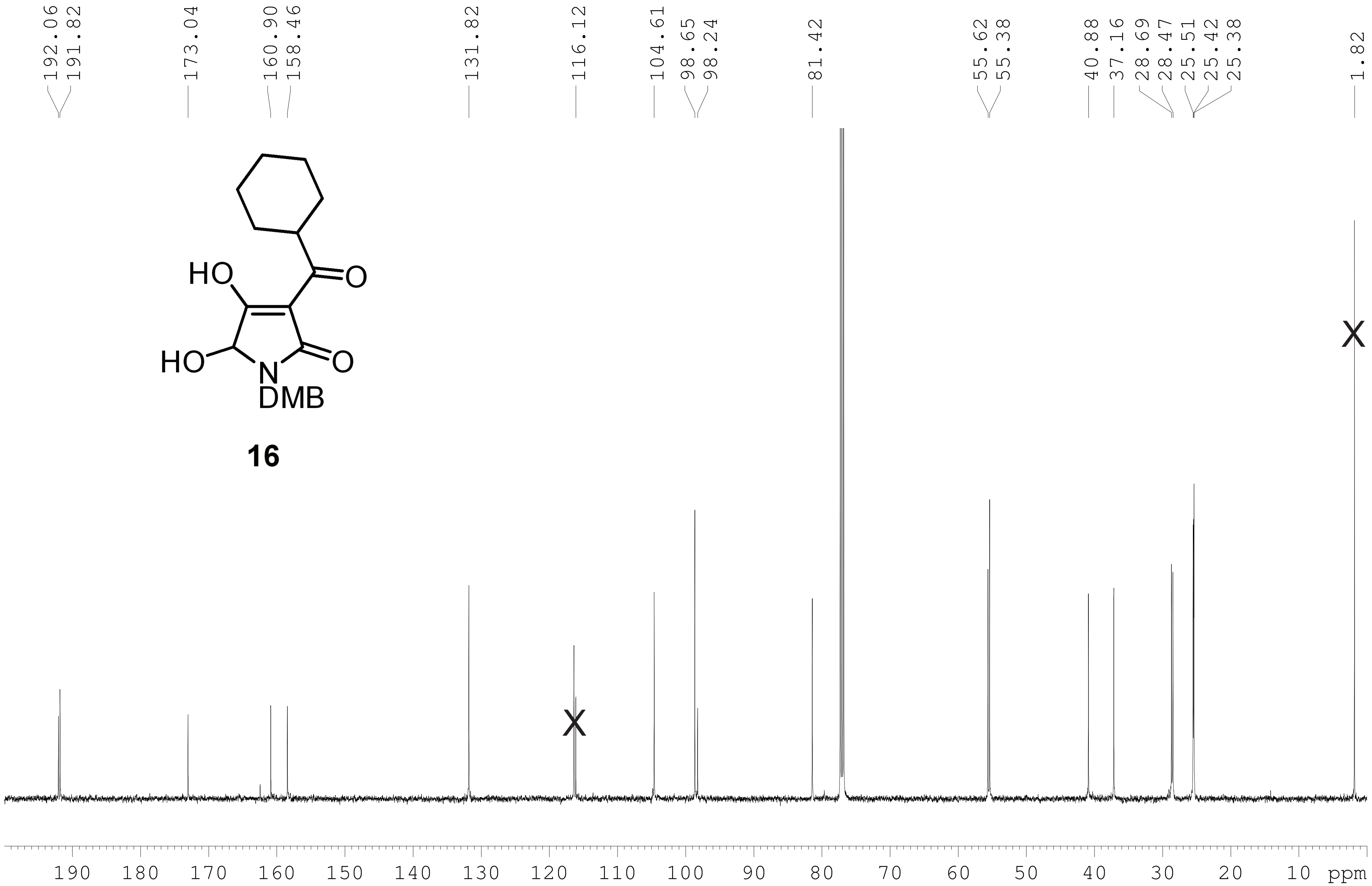
192.06
191.82
173.04
160.90
158.46
131.82
116.12
104.61
98.65
98.24
81.42
55.62
55.38
40.88
37.16
28.69
28.47
25.51
25.42
25.38
1.82
HO
HO
O
O
N
DMB
16
X
X
190 180 170 160 150 140 130 120 110 100 90 80 70 60 50 40 30 20 10 ppm

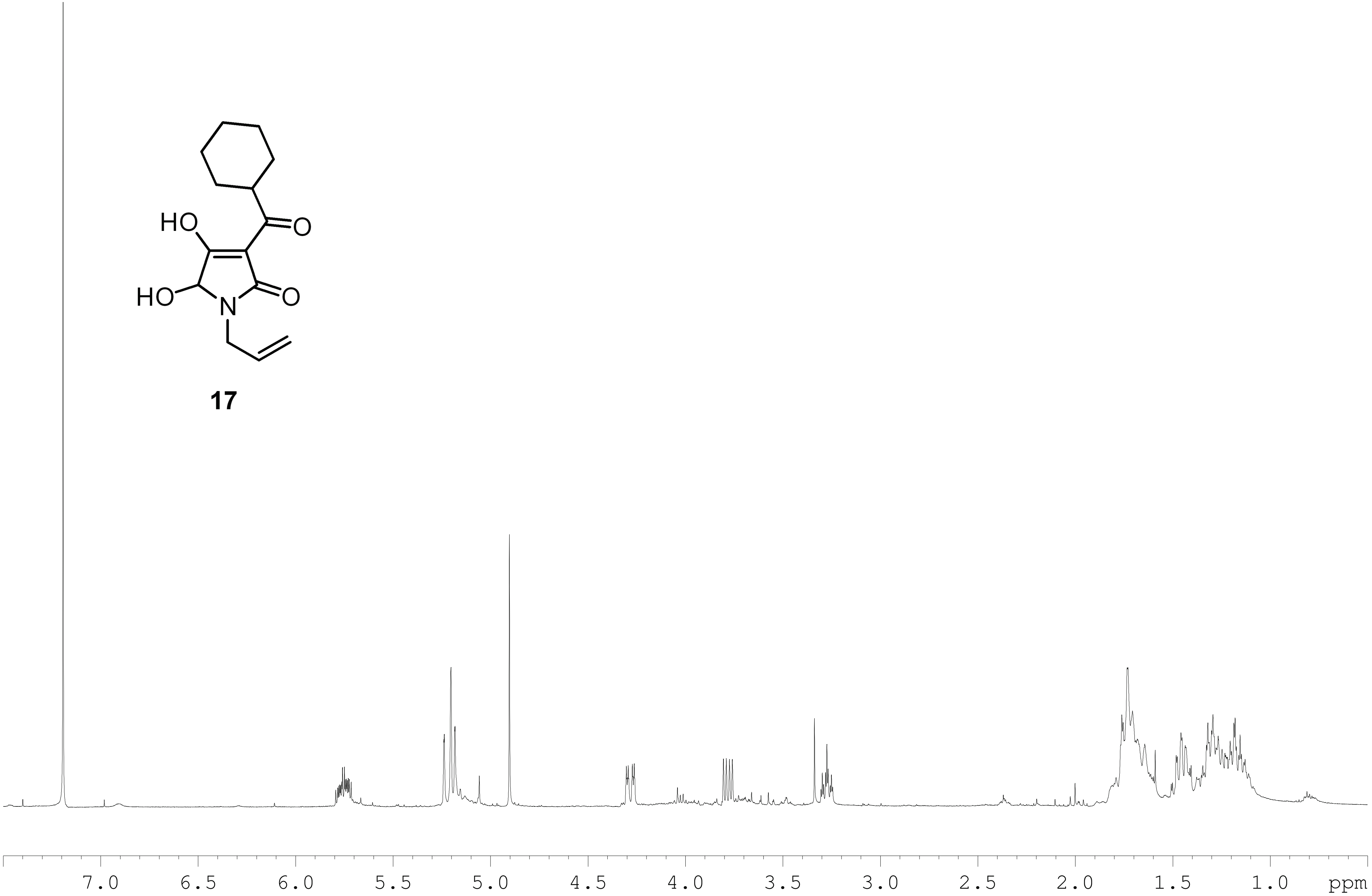
HO
HO
N
O
O
17
7.0
6.5
6.0
5.5
5.0
4.5
4.0
3.5
3.0
2.5
2.0
1.5
1.0
ppm

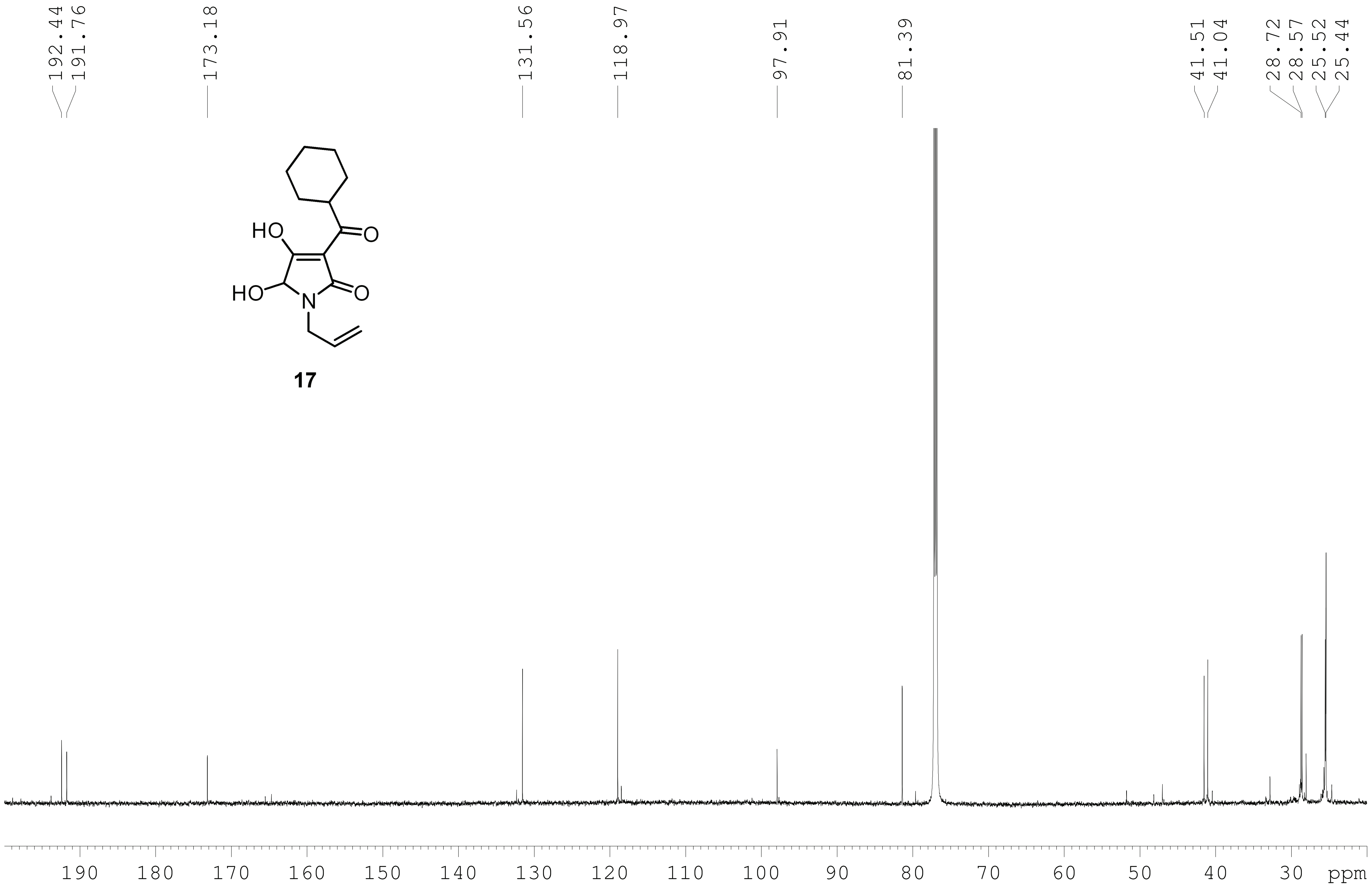
192.44
191.76
173.18
131.56
118.97
97.91
81.39
41.51
41.04
28.72
28.57
25.52
25.44
HO
HO
N
O
O
17
190 180 170 160 150 140 130 120 110 100 90 80 70 60 50 40 30 ppm

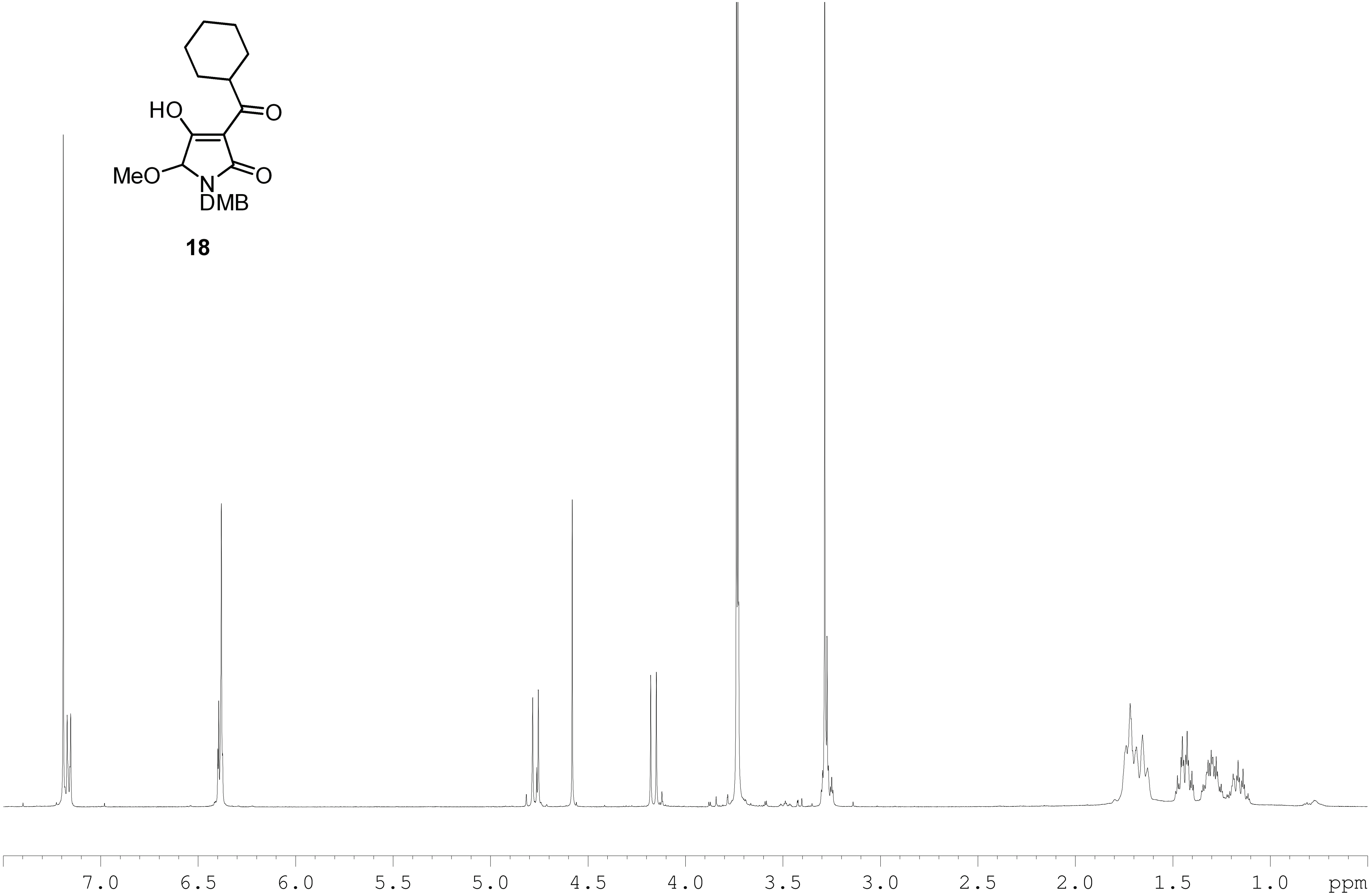

18

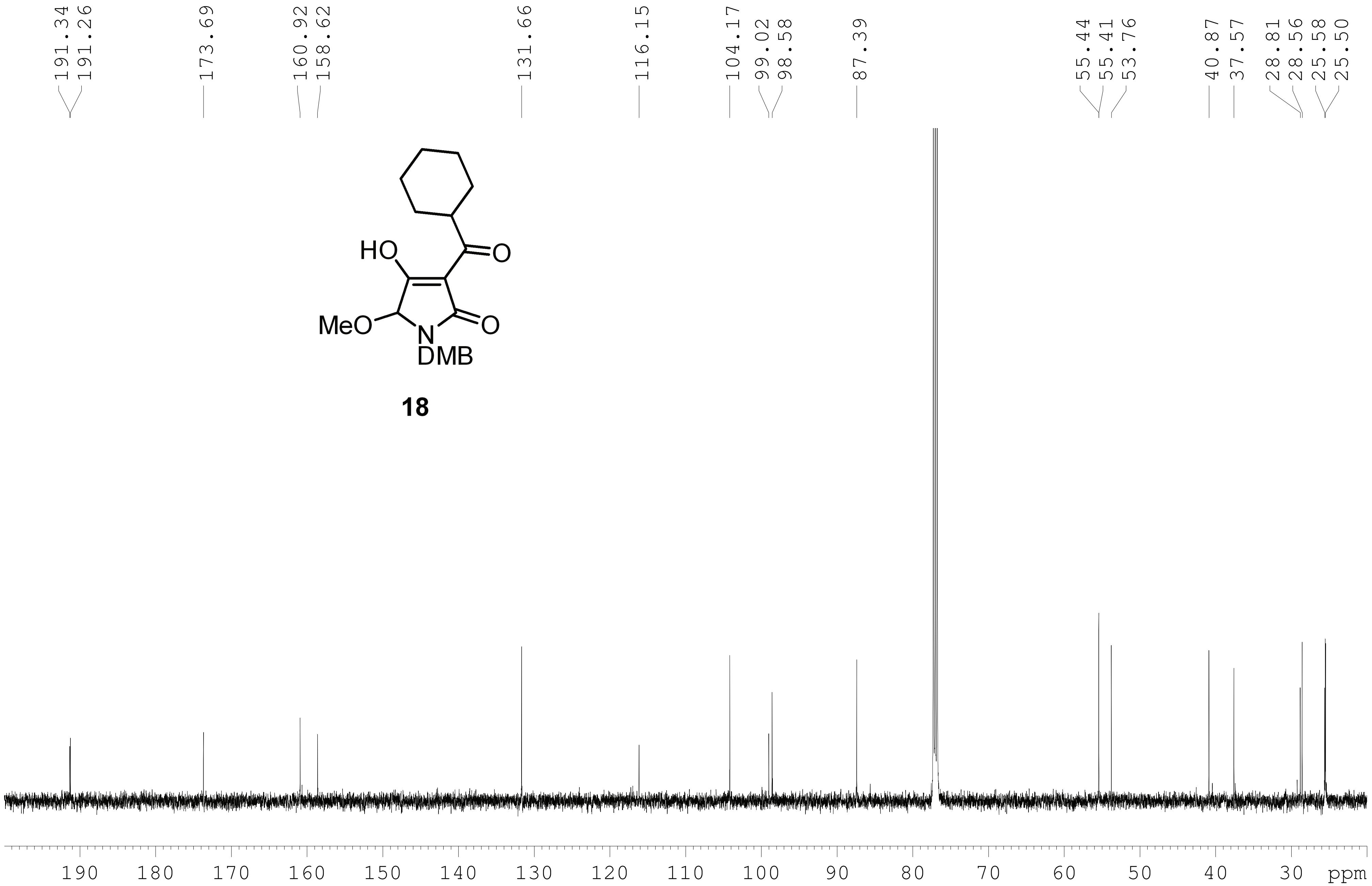
191.34
191.26
173.69
160.92
158.62
131.66
116.15
104.17
99.02
98.58
87.39
55.44
55.41
53.76
40.87
37.57
28.81
28.56
25.58
25.50
HO
O
MeO
N
O
DMB
18
190 180 170 160 150 140 130 120 110 100 90 80 70 60 50 40 30 ppm

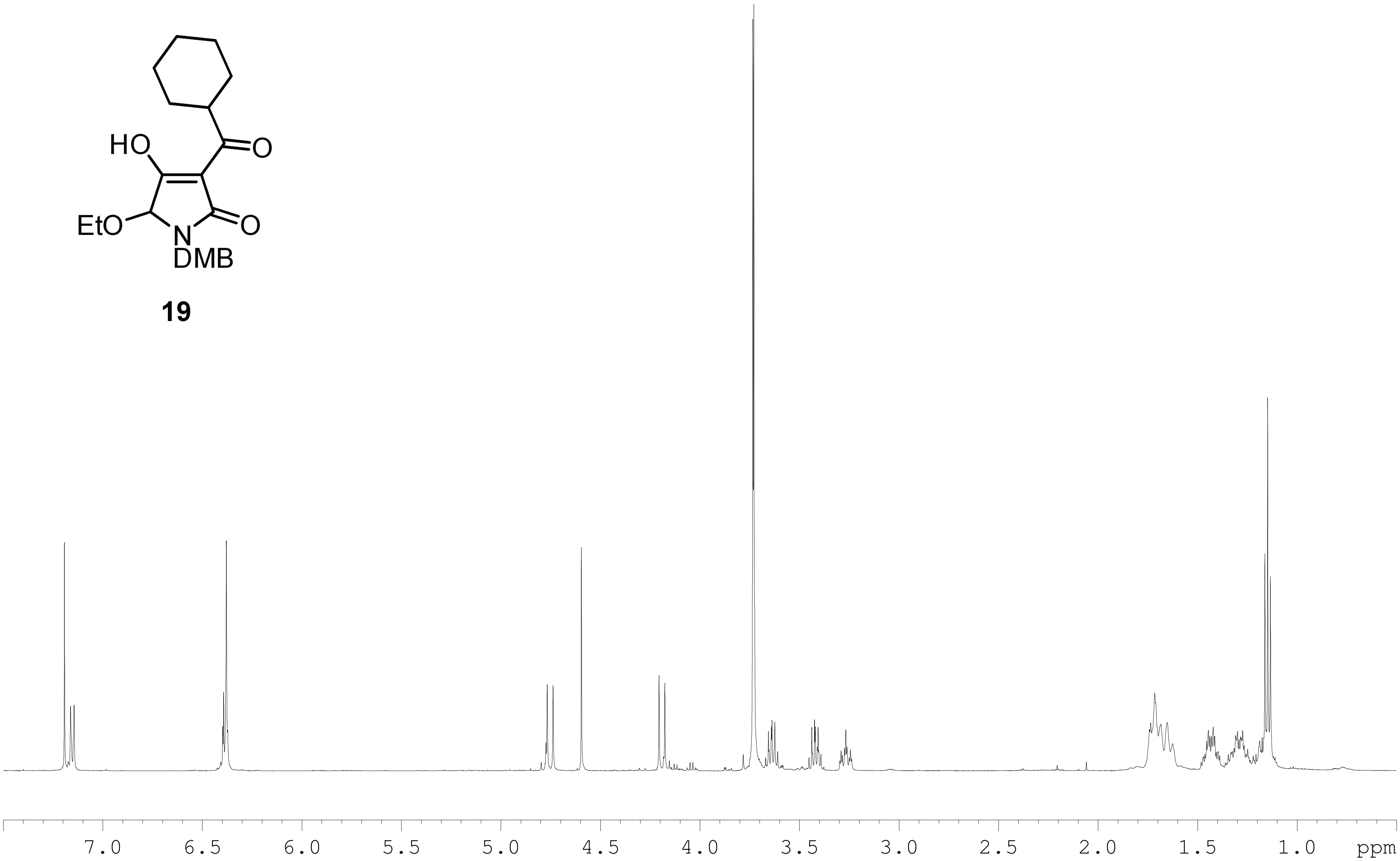
HO
O
EtO
N
O
DMB
19
7.0
6.5
6.0
5.5
5.0
4.5
4.0
3.5
3.0
2.5
2.0
1.5
1.0
ppm

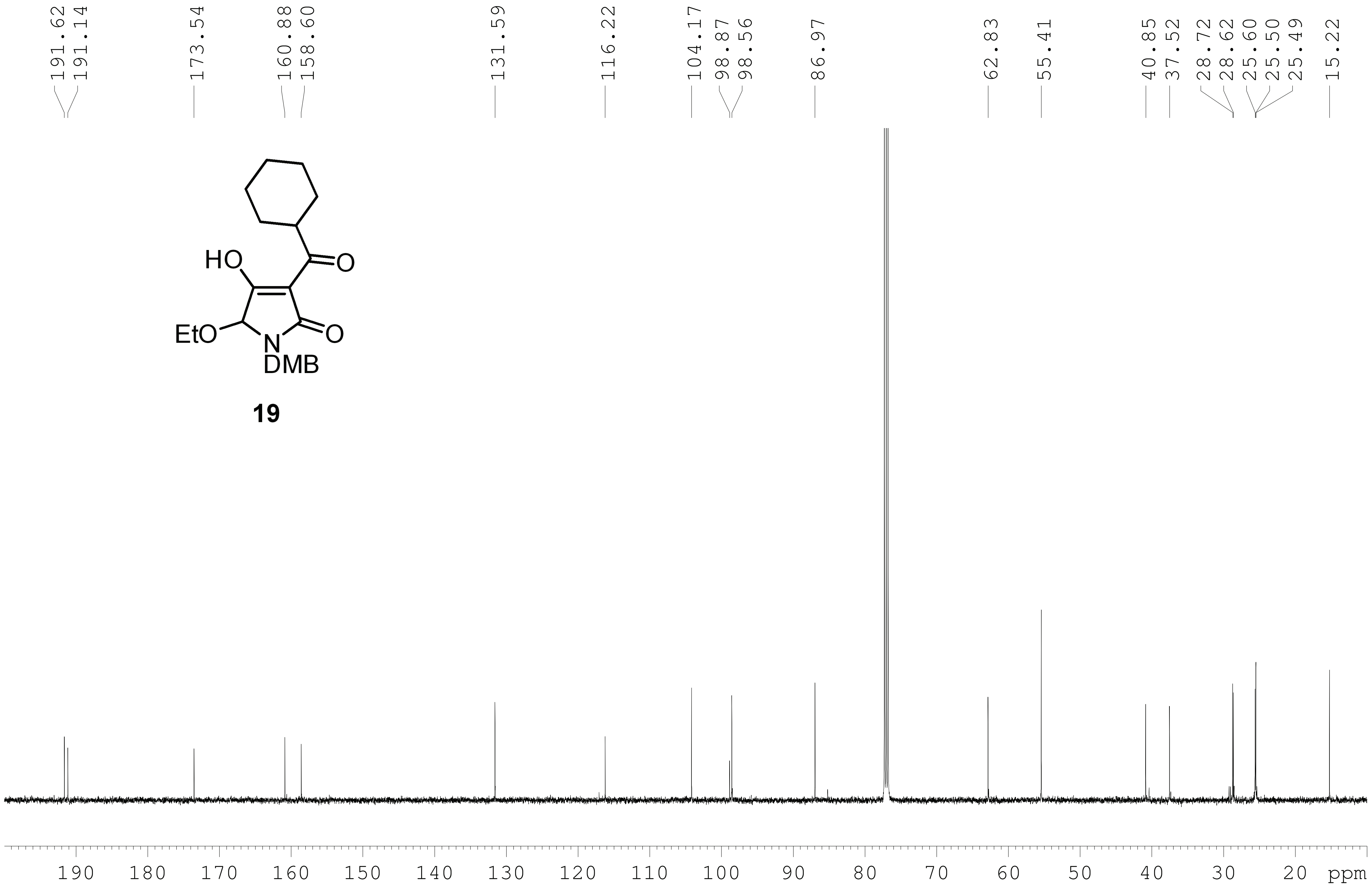
191.62
191.14
173.54
160.88
158.60
131.59
116.22
104.17
98.87
98.56
86.97
62.83
55.41
40.85
37.52
28.72
28.62
25.60
25.50
25.49
15.22
HO
O
EtO
N
O
DMB
19
190 180 170 160 150 140 130 120 110 100 90 80 70 60 50 40 30 20 ppm

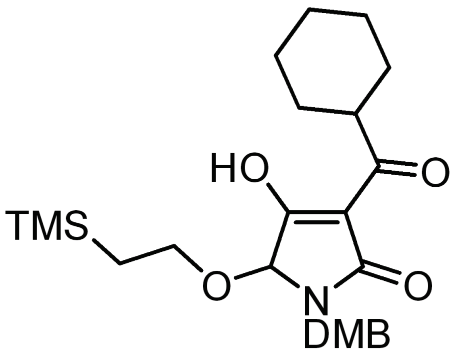

20

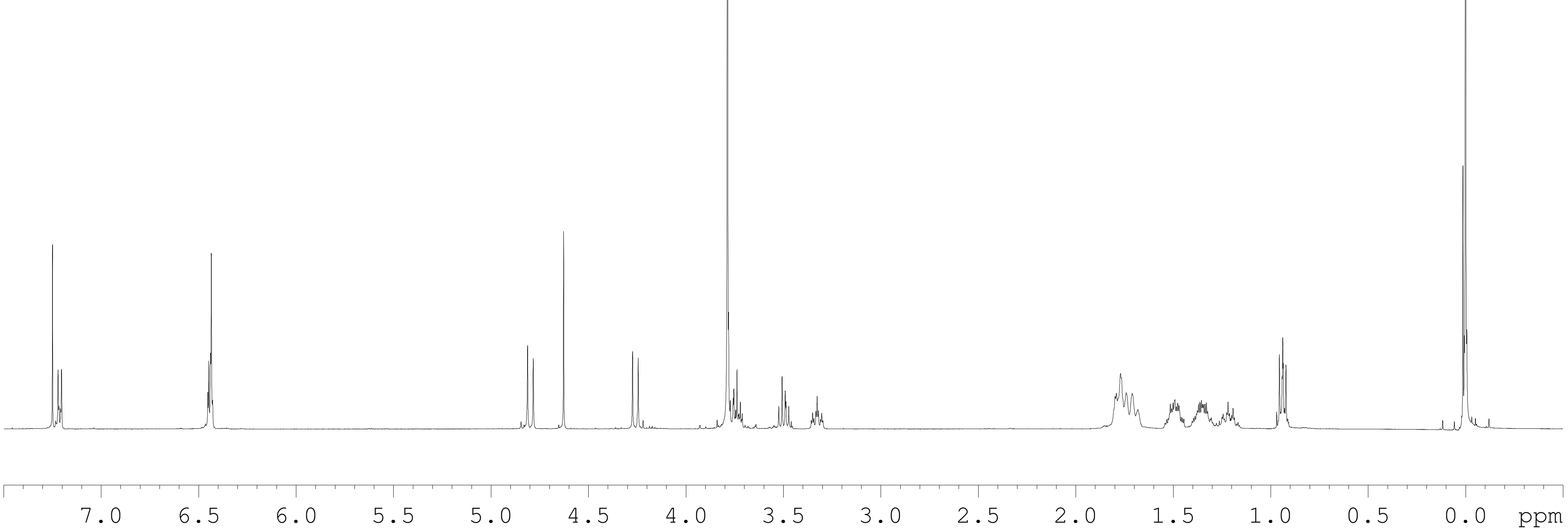

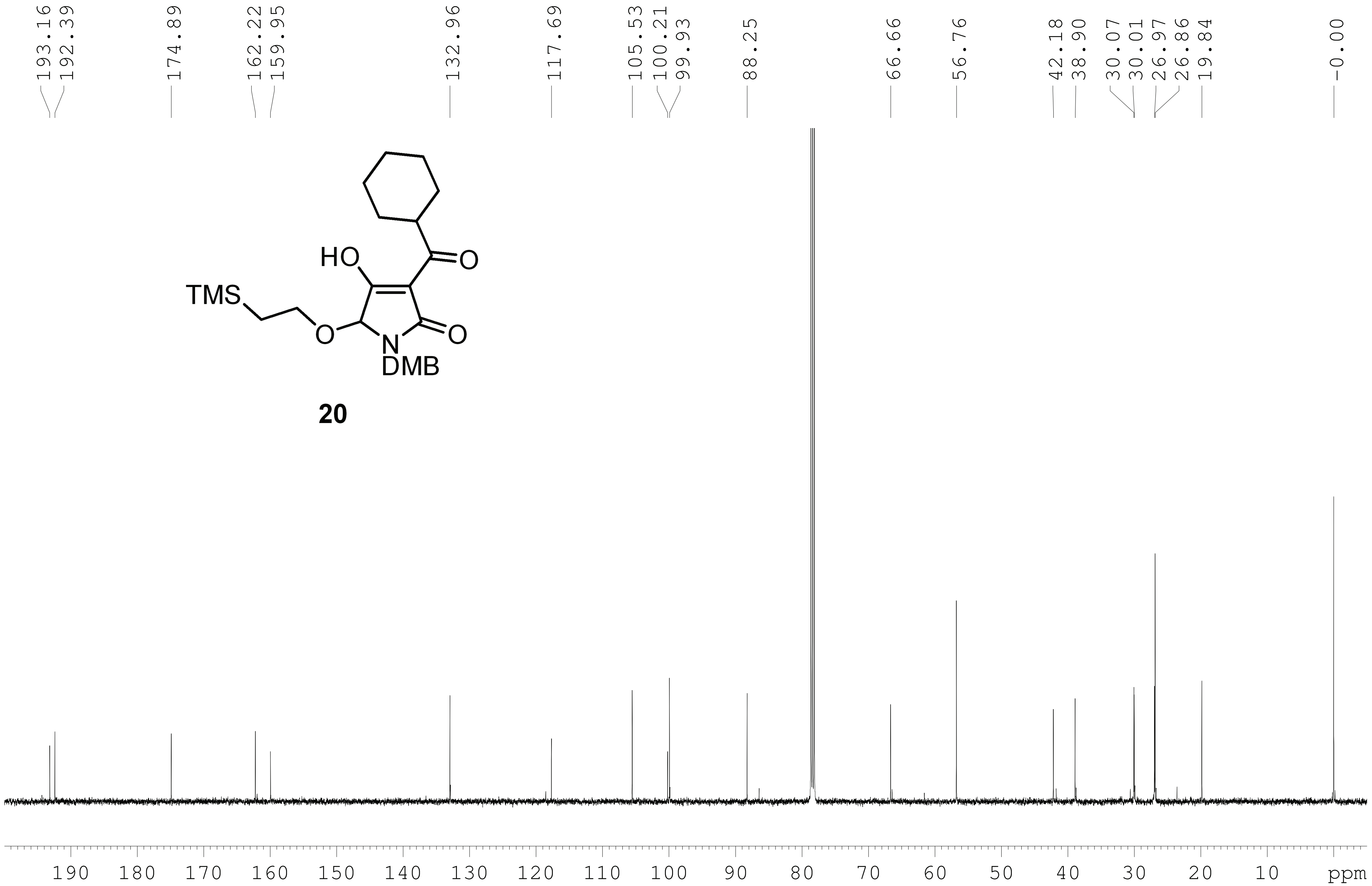
193.16
192.39
174.89
162.22
159.95
132.96
117.69
105.53
100.21
99.93
88.25
66.66
56.76
42.18
38.90
30.07
30.01
26.97
26.86
19.84
-0.00
HO
TMS
O
N
DMB
O
O
20
190 180 170 160 150 140 130 120 110 100 90 80 70 60 50 40 30 20 10 ppm

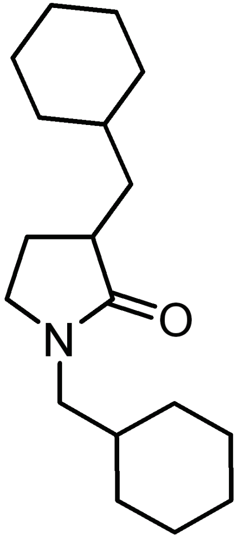

S2

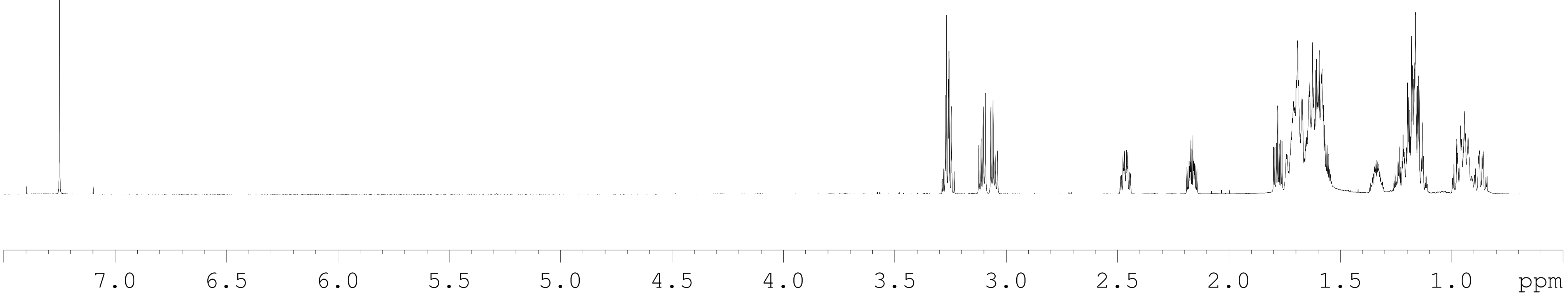

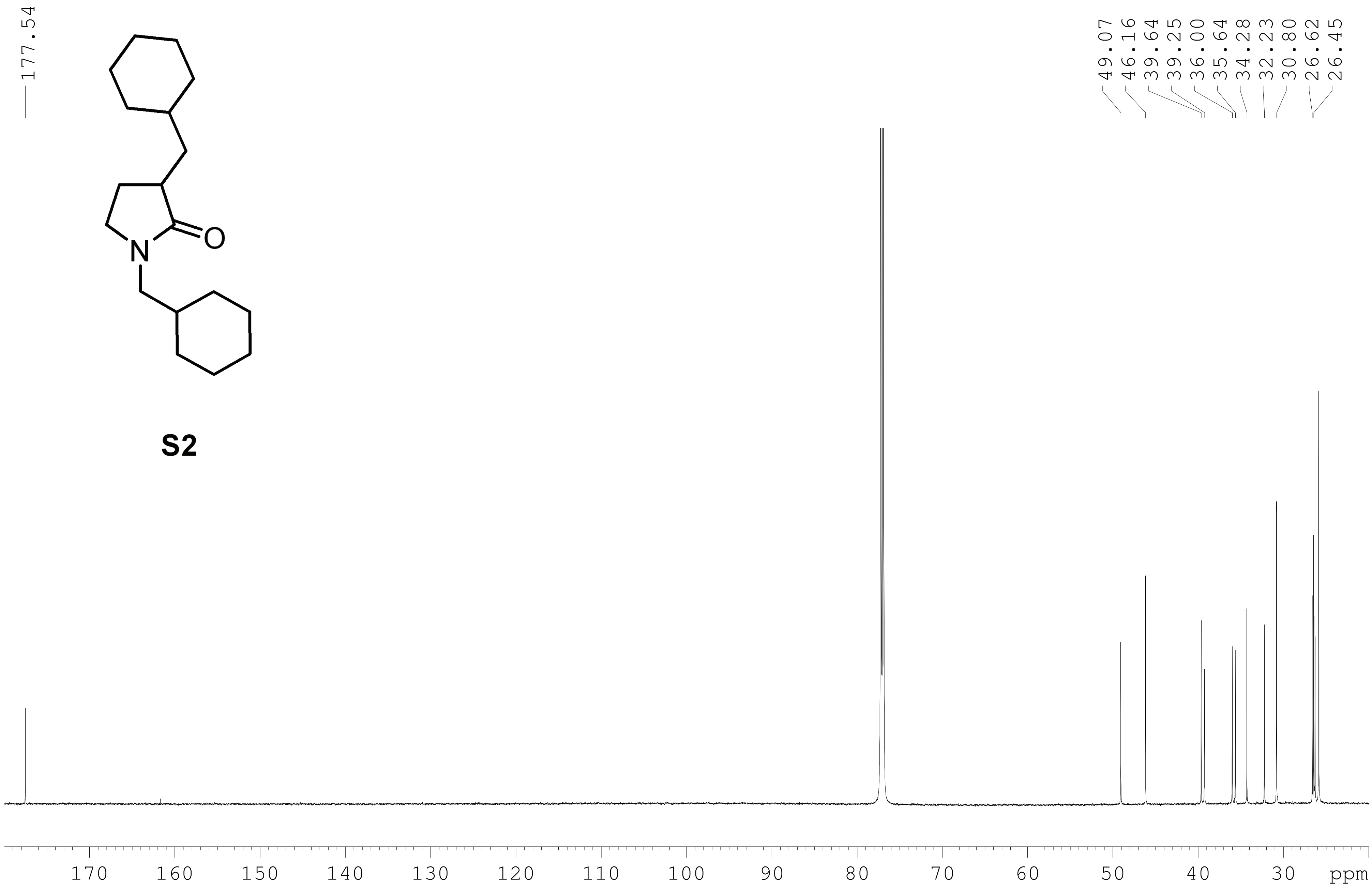
177.54
49.07
46.16
39.64
39.25
36.00
35.64
34.28
32.23
30.80
26.62
26.45
S2
170 160 150 140 130 120 110 100 90 80 70 60 50 40 30 ppm

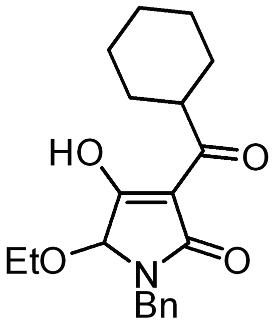

S3

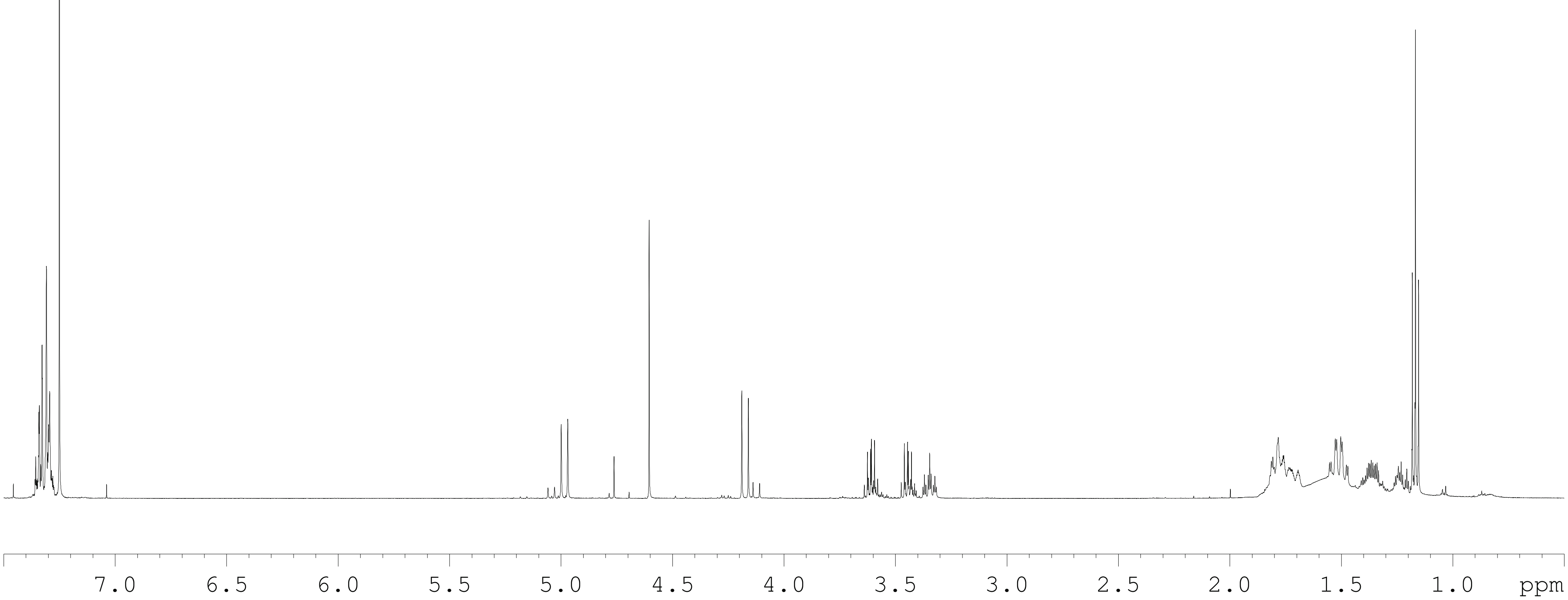

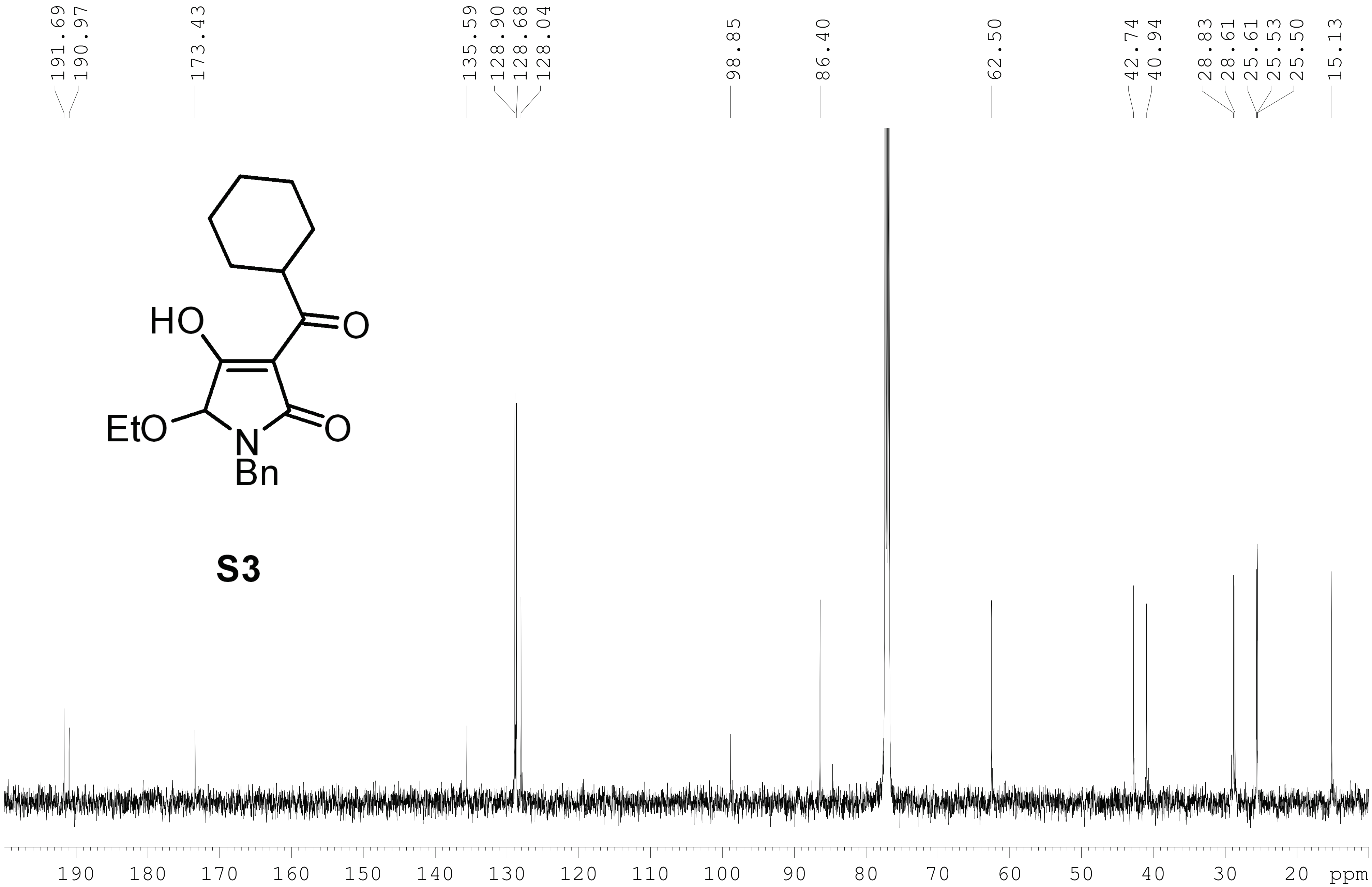
191.69
190.97
173.43
135.59
128.90
128.68
128.04
98.85
86.40
62.50
42.74
40.94
28.83
28.61
25.61
25.53
25.50
15.13
HO
O
EtO
N
Bn
O
S3
190 180 170 160 150 140 130 120 110 100 90 80 70 60 50 40 30 20 ppm

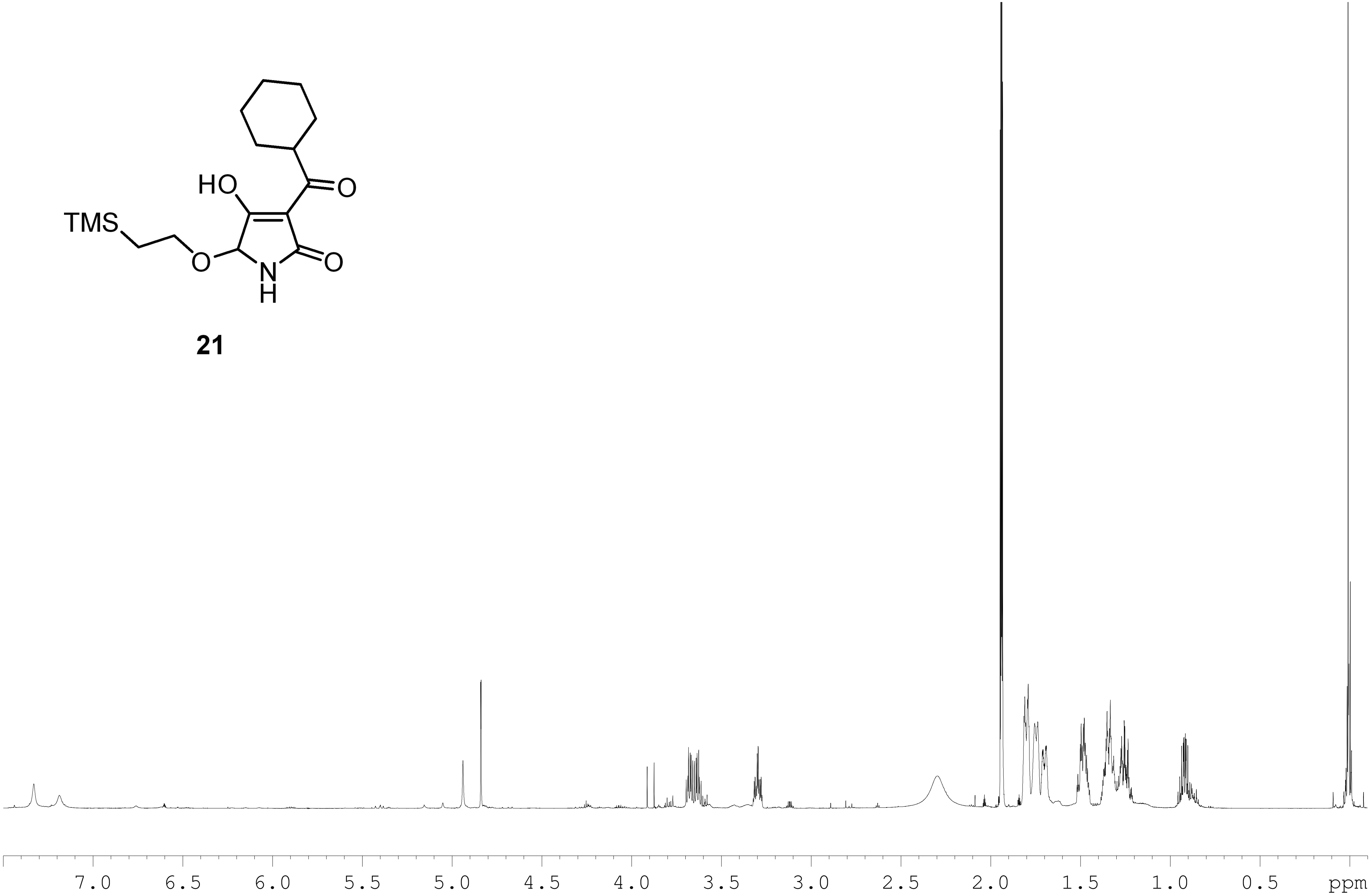

21

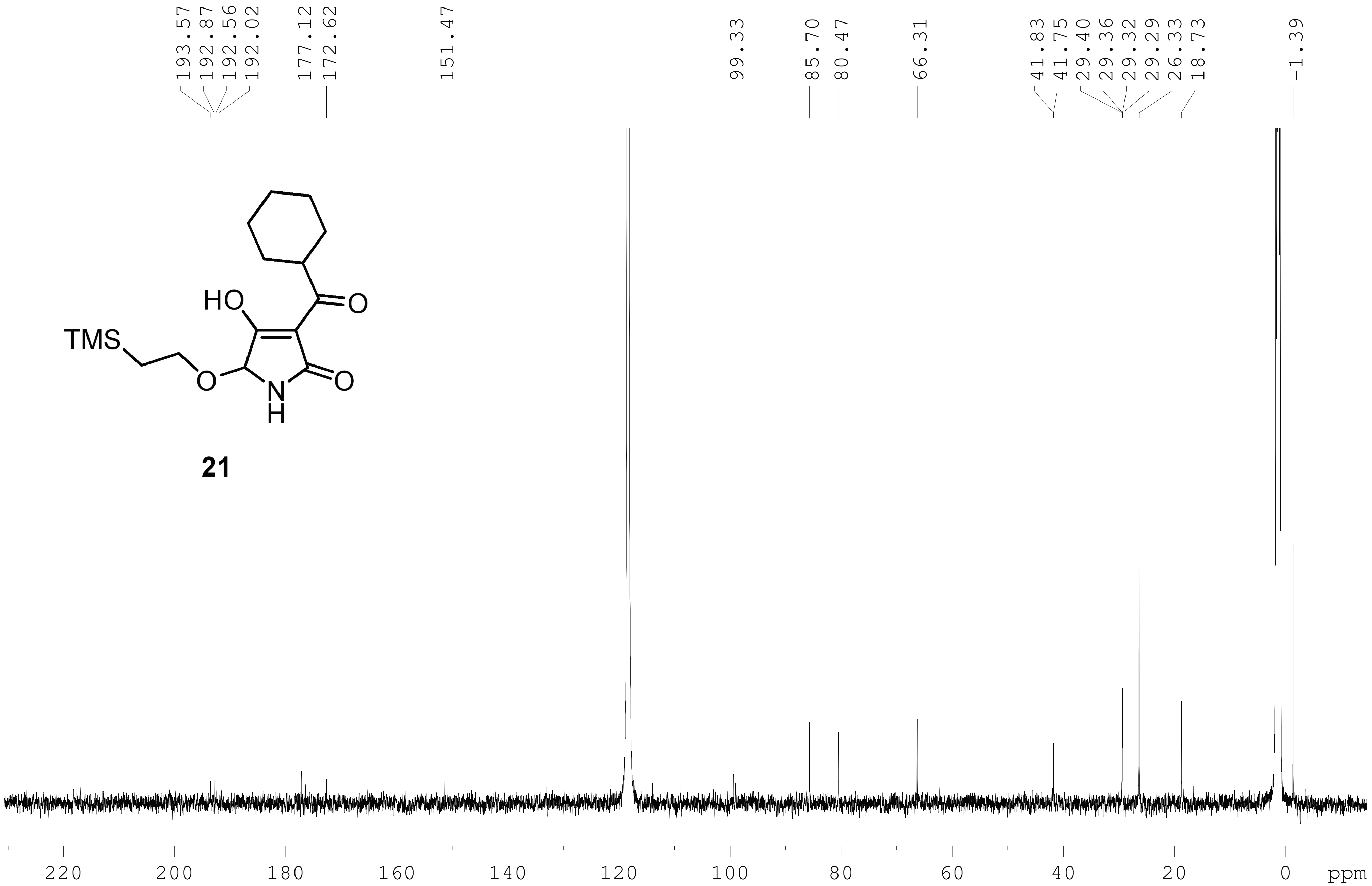
193.57
192.87
192.56
192.02
177.12
172.62
151.47
99.33
85.70
80.47
66.31
41.83
41.75
29.40
29.36
29.32
29.29
26.33
18.73
-1.39
HO
O
TMS
O
N
H
O
21
220
200
180
160
140
120
100
80
60
40
20
0
ppm

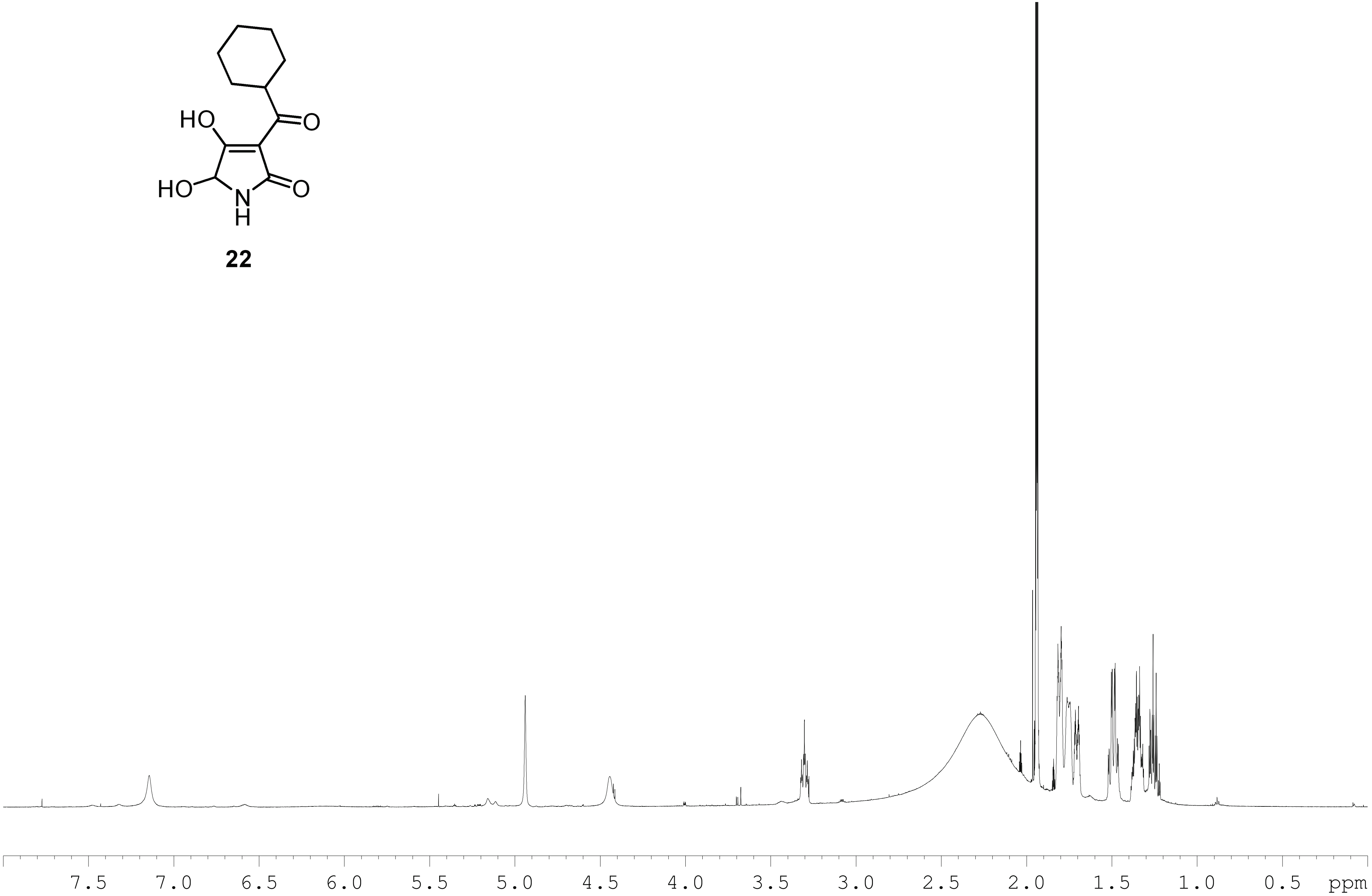

22

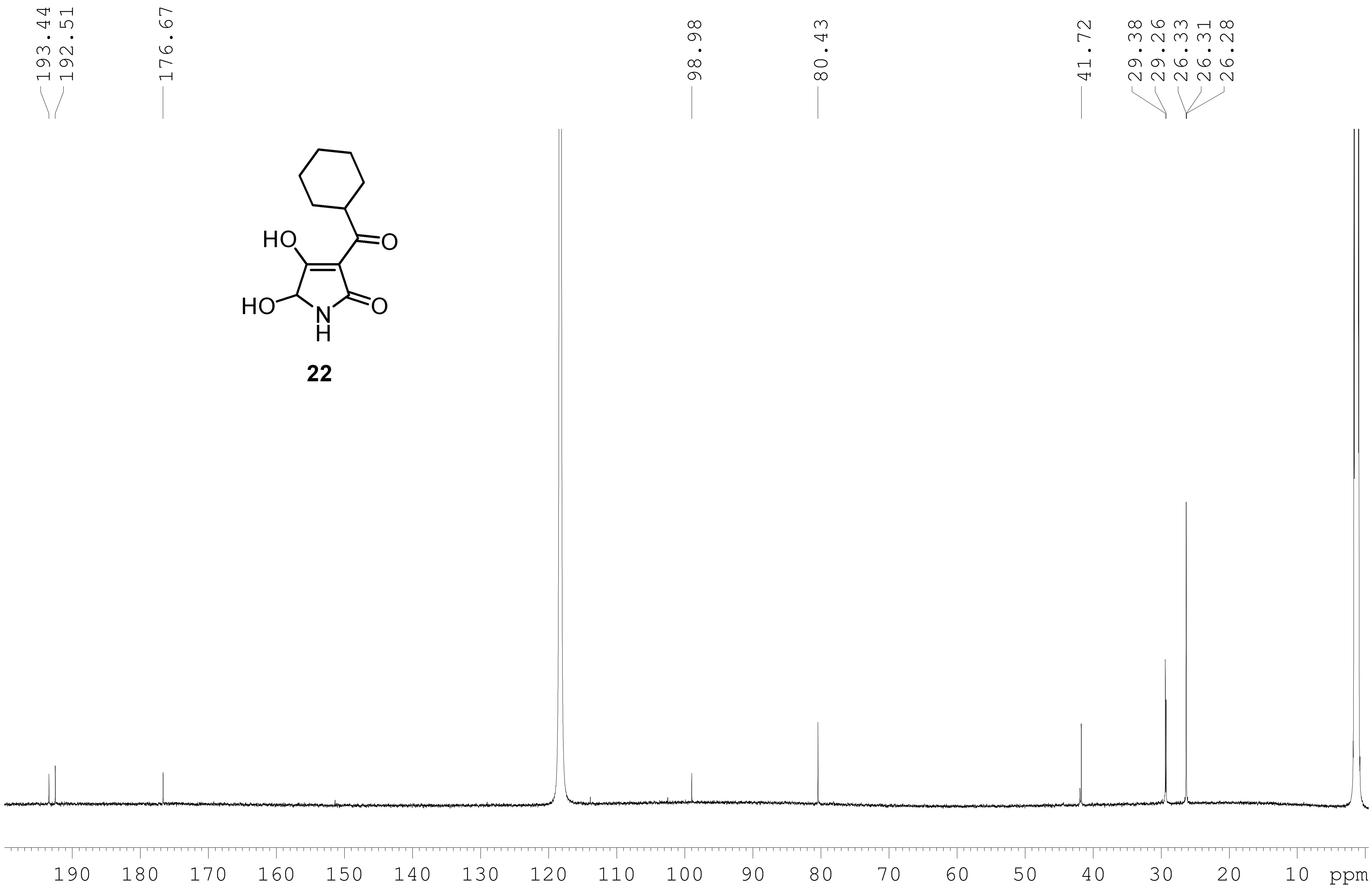
193.44
192.51
176.67
98.98
80.43
41.72
29.38
29.26
26.33
26.31
26.28
HO
O
HO
N
H
O
22
190 180 170 160 150 140 130 120 110 100 90 80 70 60 50 40 30 20 10 ppm
